# Uncovering Lasonolide A Biosynthesis Using Genome-Resolved Metagenomics

**DOI:** 10.1128/mbio.01524-22

**Published:** 2022-09-20

**Authors:** Siddharth Uppal, Jackie L. Metz, René K. M. Xavier, Keshav Kumar Nepal, Dongbo Xu, Guojun Wang, Jason C. Kwan

**Affiliations:** a Division of Pharmaceutical Sciences, School of Pharmacy, University of Wisconsin—Madison, Madison, Wisconsin, USA; b Harbor Branch Oceanographic Institute, Florida Atlantic University, Boca Raton, Florida, USA; University of Maryland School of Medicine

**Keywords:** lasonolide A, horizontal gene transfer, multiple repeats, *Verrucomicrobia*, *trans-*AT PKS, genome reduction, symbiosis

## Abstract

Invertebrates, particularly sponges, have been a dominant source of new marine natural products. For example, lasonolide A (LSA) is a potential anticancer molecule isolated from the marine sponge *Forcepia* sp., with nanomolar growth inhibitory activity and a unique cytotoxicity profile against the National Cancer Institute 60-cell-line screen. Here, we identified the putative biosynthetic pathway for LSA. Genomic binning of the *Forcepia* sponge metagenome revealed a Gram-negative bacterium belonging to the phylum *Verrucomicrobia* as the candidate producer of LSA. Phylogenetic analysis showed that this bacterium, here named “*Candidatus* Thermopylae lasonolidus,” only has 88.78% 16S rRNA identity with the closest relative, Pedosphaera parvula Ellin514, indicating that it represents a new genus. The lasonolide A (*las*) biosynthetic gene cluster (BGC) was identified as a *trans*-acyltransferase (AT) polyketide synthase (PKS) pathway. Compared with its host genome, the *las* BGC exhibits a significantly different GC content and pentanucleotide frequency, suggesting a potential horizontal acquisition of the gene cluster. Furthermore, three copies of the putative *las* pathway were identified in the candidate producer genome. Differences between the three *las* repeats were observed, including the presence of three insertions, two single-nucleotide polymorphisms, and the absence of a stand-alone acyl carrier protein in one of the repeats. Even though the verrucomicrobial producer shows signs of genome reduction, its genome size is still fairly large (about 5 Mbp), and, compared to its closest free-living relative, it contains most of the primary metabolic pathways, suggesting that it is in the early stages of reduction.

## INTRODUCTION

**FIG 1:**
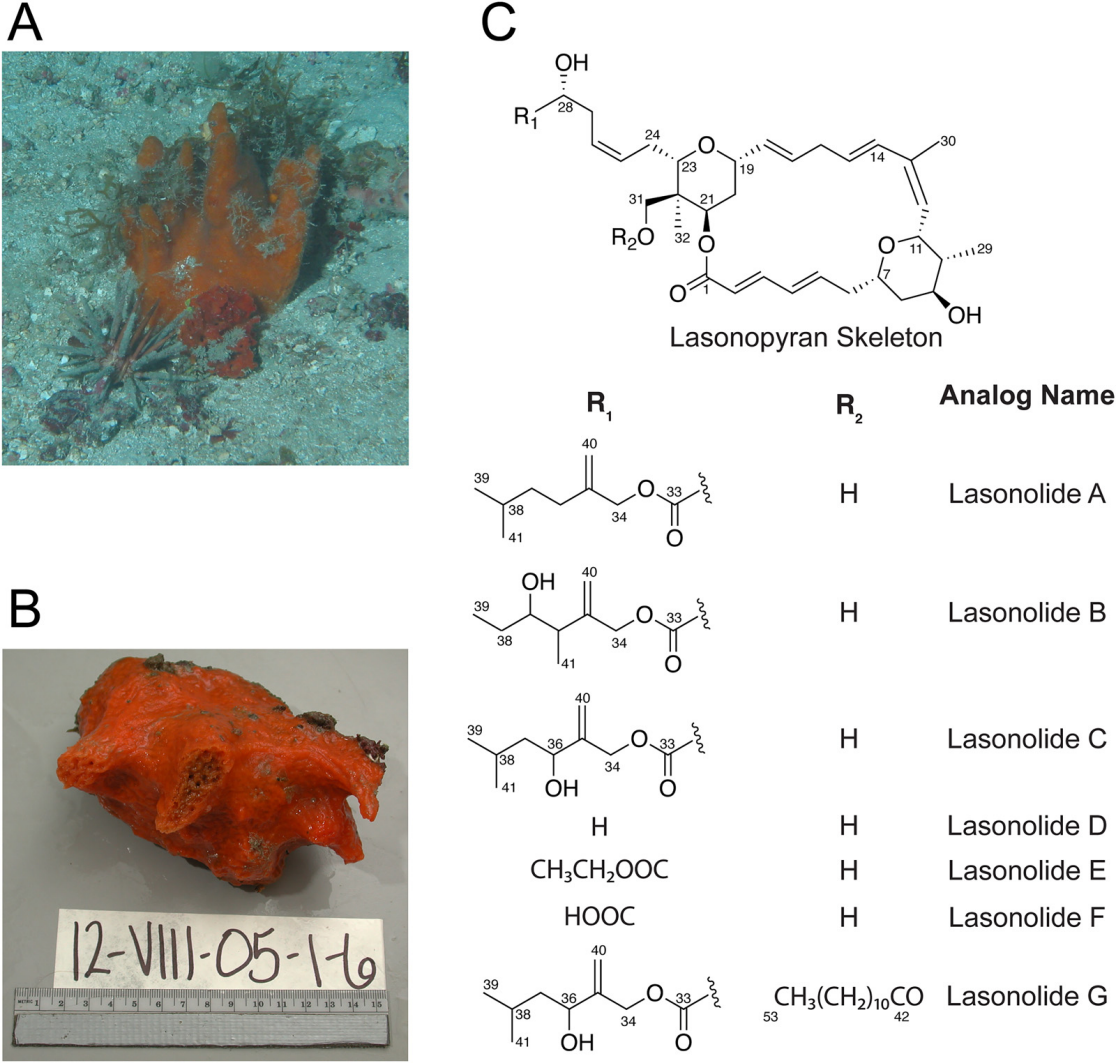
(A) Sponge *Forcepia* sp. as seen in the field. (B) *Forcepia* sp. specimen used for DNA extraction (sample ID 12-VIII-05-1-6). Photo credit: HBOI Marine Biomedical and Biotechnology Program. (C) Chemical structures of lasonolide A (LSA) and its analogs.

Lasonolide A (LSA) is a cytotoxic polyketide derived from the marine sponge *Forcepia* sp. (Fig. 1A and B) (1). Out of its analogs (B to G) (Fig. 1C), LSA is the most potent (2) and exhibits 50% inhibitory concentration (IC_50_) values in the nanomolar range against certain cell lines in the National Cancer Institute 60-cell-line screen (3). Furthermore, it has a unique mechanism of action, which includes induction of premature chromosome condensation, loss of cell adhesion, and activation of the RAF1 kinase in the Ras pathway, along with cell blebbing and contraction (3–5). This makes it a promising candidate as a scaffold for future pharmaceutical development. However, a major challenge to LSA’s clinical development is the lack of availability. Scarcity and limited accessibility of the sponge prevent it from being a sustainable source of lasonolide A. Furthermore, the chemical synthesis of LSA is tedious and has poor yields, limiting its scalability (6–8).

It is well-known that bacteria living in a symbiotic relationship with higher animals are valuable sources of novel bioactive secondary metabolites (9). In many instances, these molecules serve a protective function for the host, but the identity of the microbial producer remains unknown (9–12). Based on its potent antitumor activity, it is likely that LSA also acts as a chemical defense within its host sponge. Attempts to isolate small molecule-producing host-associated microbes are hampered by low cultivation success; it is estimated less than 1% of bacteria are currently culturable from the environment (13–15). These drawbacks have created the need to genetically engineer surrogate hosts for the sustainable and sufficient production of the desired natural products in the laboratory. The first step in engineering microbes for production of bioactive compounds is to identify the genes responsible for natural product synthesis, which can be elucidated through metagenomic analysis and cloning (16, 17). The structure of LSA very likely arises from an assembly line-type polyketide synthase (PKS) rather than the iterative PKSs that predominate in fungi and other eukaryotes, and therefore, the source is likely bacterial (18–20). Identifying the bacterium responsible for synthesizing LSA and elucidating its biosynthetic pathway will allow us to explore routes for LSA’s heterologous expression and potentially facilitate the synthesis of analogs.

Here, we describe a *trans*-acyltransferase (AT) PKS pathway (*las*) that is likely responsible for the biosynthesis of LSA. Furthermore, the entire *las* biosynthetic gene cluster (BGC) has been captured on five overlapping fosmids and reassembled for future heterologous expression. We propose that the *las* BGC is present in a yet-uncultivated bacterium belonging to a novel genus under the phylum *Verrucomicrobia*. Additionally, evidence suggests *las* BGC is repeated thrice within the symbiont with minor sequence variations between them. We also suggest that the *las* BGC has been horizontally acquired and has a codon adaptation index comparable to that of highly expressed genes. Finally, we show that the *Verrucomicrobia* symbiont is in the very early stages of genome reduction and is likely to further reduce its size.

## RESULTS AND DISCUSSION

### Identification and capture of the *las* BGC.

**FIG 2:**
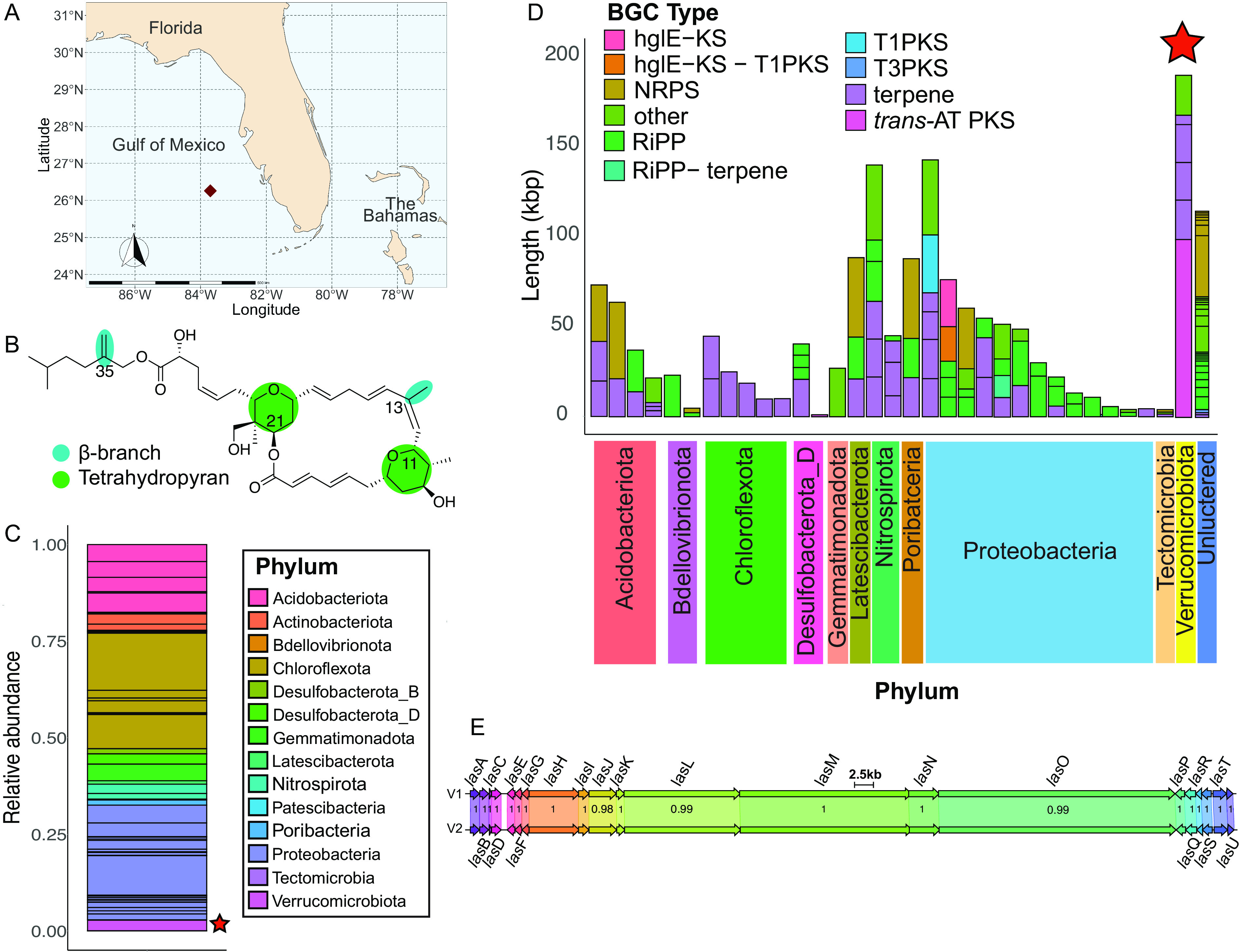
(A) Collection site of *Forcepia* sp. sponge (dark red diamond; 26.256573°N, 83.702772°W). (B) Features in lasonolide A (LSA) characteristic of biosynthesis by a *trans-*AT PKS pathway. (C) Relative abundance of different phyla (GTDB taxonomy) in the sequenced Forcepia_v1 metagenome. Each block shows the relative abundance of each metagenome-assembled genome (MAG), with colors representing the phyla they belong to. The *las* biosynthetic gene cluster (BGC)-carrying bin is marked with a star. (D) BGC distribution in *Forcepia*_v1 sp. metagenome. AntiSMASH (27) annotations of bacterial contigs greater than 500 bp are shown. Each bar indicates a metagenome-assembled genome (MAG). Bars have been grouped by phylum (GTDB taxonomy). The star marks the MAG possessing *las* BGC. BGC annotations have been simplified into polyketide synthase (PKS) type 1; PKS; type 3 PKS; *trans-*AT PKS, nonribosomal peptide synthetase (NRPS); ribosomally synthesized, posttranslationally modified peptide (RiPP); hgIE-KS; hgIE-KS-T1PKS; terpenes; RiPP-terpene; and others. (E) Comparison of *las* BGC_v1 and *las* BGC_v2 using clinker (29). V1 refers to *las* BGC_v1, while V2 refers to *las* BGC_v2. Numbers in the boxes indicate amino acid identity as a fraction of 1.

In our initial studies, we constructed a high-capacity metagenomic DNA library consisting of ~600,000 CFU from *Forcepia* sp. sponges collected from the Gulf of Mexico (Fig. 2A) to search for potential *las* biosynthetic genes. The structure of LSA contains two tetrahydropyran rings and two β-methylations (21, 22) at C-13 and C-35 (Fig. 2B). These structural features have been identified in a variety of *trans-*AT PKS pathways but are rarely found in *cis-*AT PKS systems (23, 24), thus hinting that LSA is produced by a *trans-*AT PKS pathway (24). Therefore, we screened the *Forcepia* fosmid library with clade-guided degenerate primers targeted to conserved *trans*-AT PKS genes involved in β-methylation, such as 3-hydroxy-3-methyglutaryl-CoA (HMG-CoA) synthase, free-standing ketosynthase (KS), acyl carrier protein (ACP), and two enoyl-CoA hydratases (ECH) (see Table S1A in the supplemental material). From the metagenomic library, five fosmids were identified using these primers (fosmids 5-16, 6-71, 3-46, 1-80, and 4-77), resulting in the capture of approximately 48 kb of the putative *las* BGC at its 3′ end (Fig. S1A). However, minimal progress was made toward capturing the remaining half of the BGC, as primer walking failed to produce new hits in the region upstream of fosmid 5-16. Therefore, we sequenced the metagenome of *Forcepia* sp. and searched for *trans-*AT PKS BGCs. DNA was extracted from two different regions (referred to as Forcepia_v1 and Forcepia_v2) of the same sponge and subjected to shotgun metagenomic sequencing. The reads were trimmed, assembled, and then binned into metagenome-assembled genomes (MAGs). The metagenomes were found to be abundant in *Acidobacteria*, *Proteobacteria*, and *Chloroflexota* (Fig. 2C and Fig. S1B), with 56 and 55 MAGs recovered from the two metagenomes, respectively. Based on MIMAG (25) standards for completeness and contamination, 11 and 6 MAGs were high quality, with 21 and 19 MAGs being medium quality for Forcepia_v1 and Forcepia_v2, respectively (Table S2).

10.1128/mbio.01524-22.1 FIG S1 (A) Alignment of fosmids to the *las* BGC. Fosmids are depicted as arrows above the *las* BGC. Fosmids captured before WGS are colored orange (3-46, 5-16, 6-17, 4-77, and 1-80), whereas fosmids captured after WGS are colored blue (5-41, 2-18, and 2-13). (B) Relative abundance of different phyla (GTDB taxonomy) in the sequenced Forcepia_v2 metagenome. Each block shows the relative abundance of each metagenome-assembled genome (MAG), with different colors representing the phylum they belong to. The *las* BGC-carrying bin is marked with a star. (C) Assembly graph of *las* BGC_v1 and contigs connected to it visualized in BANDAGE (R. R. Wick, M. B. Schultz, J. Zobel, K. E. Holt, Bioinformatics 31:3350–3352, 2015, https://doi.org/10.1093/bioinformatics/btv383). (D) Mapping of paired-end reads to contigs connected to *las* BGC_v1. Contigs in green boxes represent the *las* BGC, red boxes represent the 5′ end of *las* BGC, and blue boxes represent the 3′ end of *las* BGC. (E) Assembly of the seven contigs making up *las* BGC_v2. (F) Assembly graph of *las* BGC_v2 and contigs connected to it visualized in BANDAGE (R. R. Wick, M. B. Schultz, J. Zobel, K. E. Holt, Bioinformatics 31:3350–3352, 2015, https://doi.org/10.1093/bioinformatics/btv383). (G) Mapping of paired-end reads to contigs connected to *las* BGC_v2. Contigs in green boxes represent the *las* BGC, red boxes represent the 5′ end of *las* BGC, and blue boxes represent the 3′ end of *las* BGC. Panels C, D, F, and G were edited for clarity by removing contigs which had either very few paired-end read connections, were mapping to themselves, or were very small. (H) BGC distribution in the Forcepia_v2 metagenome. AntiSMASH (K. Blin, S. Shaw, K. Steinke, R. Villebro, N. Ziemert, S. Y. Lee, M. H. Medema, T. Weber, Nucleic Acid Res 47:W81–W87, 2019, https://doi.org/10.1093/nar/gkz310) annotations of bacterial contigs greater than 3,000 bp are shown. Each bar indicates a MAG, grouped by phylum (GTDB taxonomy). The star marks the MAG containing the *las* BGC. BGC annotations have been simplified into polyketide synthase (PKS); type 1 PKS; type 3 PKS; *trans-*AT PKS; nonribosomal peptide synthetase (NRPS); ribosomally synthesized, posttranslationally modified peptide (RiPP); hgIE-KS; hgIE-KS-T1PKS; terpenes; and others. (I) Phylogenetic tree of 51 different *Verrucomicrobia* genomes. Bootstrap values were calculated using RaxML with 1,000 bootstrap replicates. Download FIG S1, EPS file, 2.9 MB. Copyright © 2022 Uppal et al. 2022 Uppal et al. https://creativecommons.org/licenses/by/4.0/ This content is distributed under the terms of the Creative Commons Attribution 4.0 International license.

10.1128/mbio.01524-22.6 TABLE S1 List of oligonucleotide primers used for different purposes. (A) Primers used for screening the *Forcepia* sp. fosmid library before WGS. (B) Primers used for confirming the presence of terminal connections with the *las* BGC. (C) Primers used for screening the *Forcepia* sp. fosmid library after WGS. (D) Primers used for DNA assembly and validation of transformants. Download Table S1, XLSX file, 0.1 MB. Copyright © 2022 Uppal et al. 2022 Uppal et al. https://creativecommons.org/licenses/by/4.0/ This content is distributed under the terms of the Creative Commons Attribution 4.0 International license.

10.1128/mbio.01524-22.7 TABLE S2 Metadata and taxonomic classification of all the MAGs. Download Table S2, XLSX file, 0.06 MB. Copyright © 2022 Uppal et al. 2022 Uppal et al. https://creativecommons.org/licenses/by/4.0/ This content is distributed under the terms of the Creative Commons Attribution 4.0 International license.

A tBLASTN (26) search of KS domains from publicly available *trans-*AT PKS pathways against our assembled metagenome was performed. In the case of Forcepia_v1, the top hits were all to a contig of length 98 kbp labeled gnl|UoN|bin5_1_edit_8, strongly suggesting that this contig contains *trans-*AT PKS genes and may possess the potential LSA pathway. Contig gnl|UoN|bin5_1_edit_8 was manually inspected and corrected for sequence gaps (Text S1). With the exception of a 1.1-kbp contig annotated as containing a *trans*-AT PKS pathway with a truncated condensation domain (in bin3674_131), analysis of the metagenome using AntiSMASH (27) (Fig. 2D) did not reveal any other BGC with plausible size or genes for the synthesis of LSA. Contig gnl|UoN|bin5_1_edit_128 (3.6 kbp) was found to be connected to the 5′ end of gnl|UoN|bin5_1_edit_8 (see “Multiple repeats of the *las* BGC” below); it encoded a stand-alone ACP domain and about 47 amino acid residues, which completed the terminal KS domain of gnl|UoN|bin5_1_edit_8. Both of these contigs were assembled together, and annotation of genes and biosynthetic domains within this assembly reaffirmed that they are likely involved in LSA synthesis, through the gene cluster we termed *las* BGC_v1. The sequence of *las* BGC_v1 was also in alignment with previously sequenced fosmids identified from the metagenomic library.

10.1128/mbio.01524-22.10 TEXT S1 Supplemental methods. Download Text S1, DOCX file, 0.03 MB. Copyright © 2022 Uppal et al. 2022 Uppal et al. https://creativecommons.org/licenses/by/4.0/ This content is distributed under the terms of the Creative Commons Attribution 4.0 International license.

Inspection of the MAGs revealed that bin5_1_edit_8 was binned with genome bin75_1. However, to our surprise, visual inspection of the assembly graph (Fig. S1C) in BANDAGE (28) indicated that bin5_1_edit_8 is present between contigs belonging to bin5_1 (phylum *Verrucomicrobia*). Furthermore, mapping paired-end reads onto bacterial contigs (Fig. S1D) showed that multiple-read pairs aligned across the junction of bin5_1_edit_8 and bin5_1. The terminal connections between contig bin5_1_edit_8 and several contigs in bin5_1 were verified via PCR (Table S1B) and Sanger sequencing of the amplicons using metagenomic DNA as the template. Based on this evidence, bin5_1_edit_8 was manually placed with bin5_1, as well as some additional contigs (Text S1).

In the case of Forcepia_v2, tBLASTN searches of *trans*-AT KS domains produced hits in eight different contigs, which could be assembled together through sequence overlaps in Geneious (https://www.geneious.com) (Fig. S1E). Except for contig bin4_1_edit_10, the other seven contigs assembled into a single large contig of 102 kbp (termed *las* BGC_v2). Similar to *las* BGC_v1, inspection of the assembly graph (Fig. S1F) and mapping of paired-end reads (Fig. S1G) revealed that contigs forming *las* BGC_v2 have been binned incorrectly and should be part of bin4_1 (phylum *Verrucomicrobia*). As a result, the contigs comprising *las* BGC_v2, as well as additional contigs (Text S1), were manually placed with bin4_1. No other contig containing a *trans*-AT PKS pathway was identified in the metagenome (Fig. S1H).

Alignment of *las* BGC from both Forcepia_v1 and Forcepia_v2 using clinker (29) revealed that these pathways are highly similar (Fig. 2E). The amino acid identity is 100% for most of the genes except for *lasJLO*, where it is 98.37%, 99.84%, and 99.83%, respectively. The slightly lower identity of *lasJLO* is due to an insertion sequence present in *las* BGC_v2 but absent in *las* BGC_v1. These insertion variants were later identified to be present in some repeats of *las* BGC_v1 as well. Interestingly, network analysis with BiG-SCAPE (30) revealed no shared families with MIBiG reference BGCs, indicating the novelty of the *las* BGC.

In order to capture the whole of the *las* BGC, a screening strategy was developed for isolating the previously missing 5′ end of the pathway from the metagenomic library using specific PCR primers. This resulted in the identification of fosmids 5-41, 2-18, and 2-13 (Fig. S1A and Table S1C), which enabled us to capture the *las* BGC minimally on 5 fosmids, 5-41, 2-18, 2-13, 5-14, and 4-71. The five fosmids were then assembled into a single vector using a newly developed CRISPR-Cas9 technology by Varigen Biosciences (Madison, WI) for future heterologous expression of the *las* BGC.

The putative symbiont genome carrying the *las* BGC (Forcepia_v1 bin5_1 and Forcepia_v2 bin4_1) was identified to belong to phylum *Verrucomicrobiota*, order *Pedosphaerales*, and genus UBA2970 by GTDB-TK v1.5.0 (database r202) (31). Excluding the *las* genes, the average nucleotide identity (ANI) of Forcepia_v1 bin5_1 and Forcepia_v2 bin4_1 is 99.9%, suggesting little strain heterogeneity between the sites in the sponge beyond a small amount perhaps attributable to sequencing errors. To our knowledge, this is the first time a *trans*-AT PKS BGC has been reported in an organism belonging to the order *Pedosphaerales*. A phylogenetic tree of 51 different *Verrucomicrobia* genomes (Fig. S1I) placed the LSA producer in subdivision 3 (NCBI taxonomy). The closest relative of the symbiont with a publicly available genome is Pedosphaera parvula Ellin514 (NCBI assembly accession no. GCA_000172555), with 88.78% identity to the 16S rRNA sequence. As per the 16S rRNA gene identity cutoffs proposed by Yarza et al. (32), this represents a new genus within the family AAA164-E04 (as classified by GTDB-Tk [31]). We named the bacterium “*Candidatus* Thermopylae lasonolidus”: Thermopylae is a tribute to the 300 Spartan hoplites and other Greek soldiers that fought at the Battle of Thermopylae. The Spartans fought to protect Greece from Persians, and the LSA-producing bacterium, with its three copies of the *las* BGC (see below), is proposed to be protecting the host sponge from predators. Lasonolidus suggests the bacterium is associated with lasonolide A and also rhymes with the Spartan king of the 300 hoplites, Leonidas. Despite being the putative producer of LSA, “*Ca.* Thermopylae lasonolidus” is not highly abundant in the metagenome, having a relative abundance of just over 2.65% in Forcepia_v1 and 1.78% in Forcepia_v2 (Fig. 2C and Fig. S1B).

### Model for lasonolide biosynthesis by *las* BGC.

**FIG 3:**
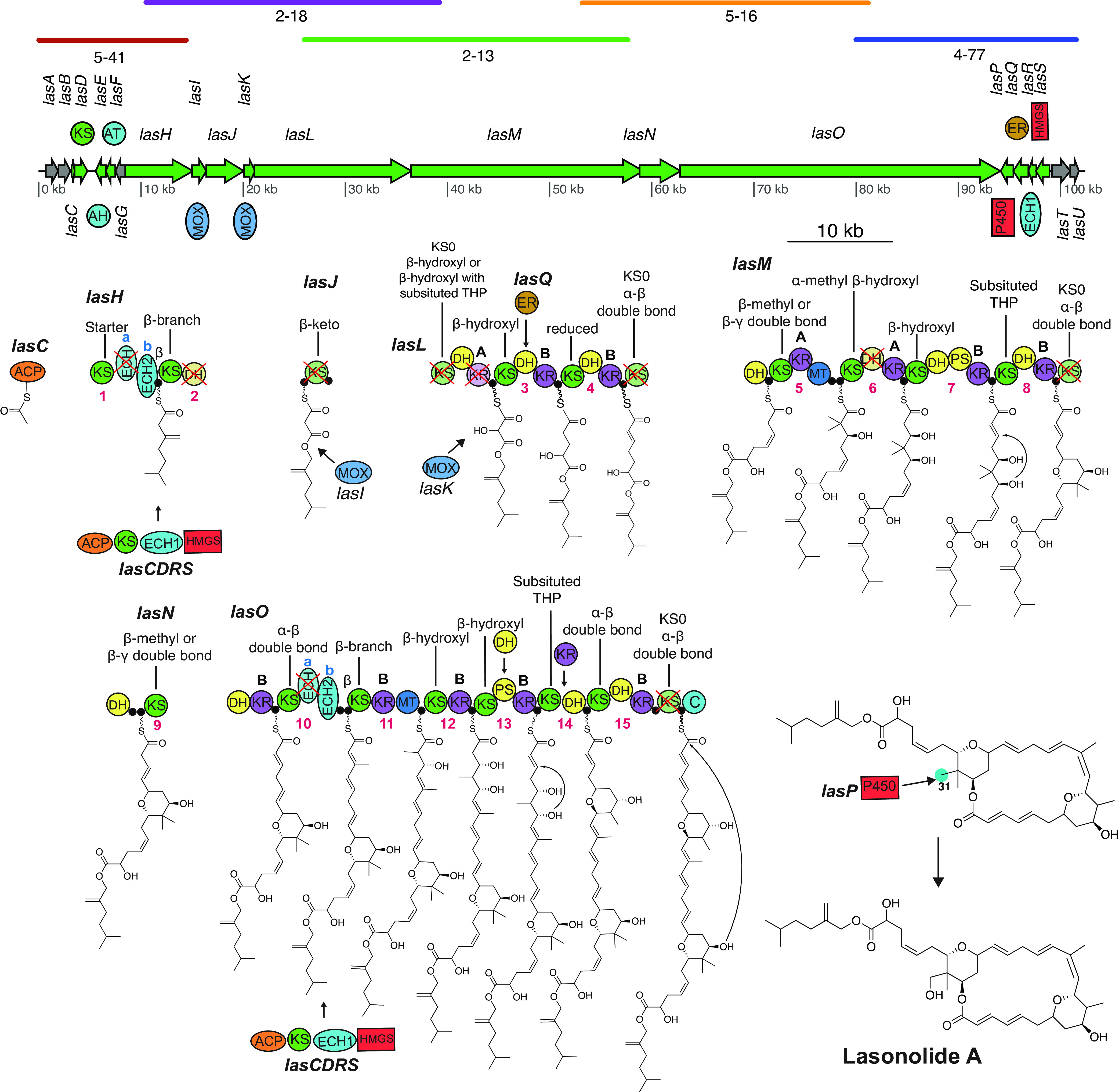
Proposed LSA biosynthetic scheme. Colored lines above the *las* BGC represent alignment of individual fosmids to the pathway. A cross indicates a domain predicted to be catalytically inactive. Open reading frames colored in gray represent genes with unknown or no role in LSA synthesis. Numbers below domains indicate the module number, and “A” and “B” denote the predicted stereoconfiguration of the KR product, as previously described (47, 48). Predicted substrate specificity of KS domains, obtained through phylogeny (Data Set S1 in the supplemental material) (33), are shown above each respective KS domain. C-31 is highlighted in blue to represent the site where P450 LasP is predicted to act. Abbreviations: ACP, acyl carrier protein, also denoted by a filled black circle; AH, acylhydrolase; AT, acyltransferase; C, condensation; DH, dehydratase; ECH, enoyl-CoA reductase; ER, enoylreductase; HMGS, 3-hydroxy-3-methylglutaryl-CoA synthase; KR, ketoreductase; KS, ketosynthase; MOX, monooxygenase; PS, pyransynthase; P450, cytochrome P450; THP, tetrahydropyran.

The proposed biosynthetic scheme for the synthesis of LSA by the *las* BGC is shown in Fig. 3. The complete *las* BGC consists of 6 *trans*-AT PKS proteins (*lasHJLMNO*), 10 accessory genes (*lasCDEFIKPQRS*), and 5 genes with no or an unknown role in LSA synthesis (*lasABGTU*). Phylogenetic analysis of 944 different KS domains (Data Set S1) was used to predict KS substrate specificity (33), and these predictions were found to be similar to the proposed biosynthetic model. The pathway is predicted to be colinear with the first KS domain of *lasH* clustering into the same clade as other starter KS domains in the KS phylogenetic tree. Moreover, the last *trans-*AT PKS protein (*lasO*) contains a condensation domain, similar to those found in nonribosomal peptide synthetase pathways, as its terminal domain. We propose this terminal condensation domain is responsible for cyclizing and cleaving the final PKS product (24).

10.1128/mbio.01524-22.3 DATA SET S1 Clades from a phylogenetic tree of 944 KS domains from *trans-*AT PKS and the erythromycin BGC as an outgroup, containing KS domains from the *las* BGC. The color within the individual clades corresponds to the chemical structure shown on its right. “i” and “c” in *lasD* KS1 indicate the incomplete and complete KS domains in *lasD,* respectively. Download Data Set S1, EPS file, 3.3 MB. Copyright © 2022 Uppal et al. 2022 Uppal et al. https://creativecommons.org/licenses/by/4.0/ This content is distributed under the terms of the Creative Commons Attribution 4.0 International license.

An acylhydrolase (AH) domain is often used in *trans*-AT PKS systems for proofreading by cleaving the acyl units from stalled sites (34, 35). AHs are closely related to acyltransferase (AT) domains, which are involved in the addition of malonyl-*S*-coenzyme A extender units on the phosphopantetheine arms of ACP domains (24, 36). LasE and LasF are identified as AH and AT domains, respectively, based on the presence of active-site residues (Data Set S2A) and phylogeny (35) (Data Set S2B). The accessory proteins LasCDRS include enzymes involved in β-branch formation at modules 1 and 10 (21). The ACPs in those modules contain a conserved tryptophan known to interact with β-branching enzymes (37, 38). LasR is proposed to be responsible for dehydration (ECH1), while LasH ECHb and LasO ECHb are responsible for decarboxylation (ECH2) during β-branch formation (39) (Data Set S2C). Due to their truncated size and lack of homology to the conserved sites needed for oxyanion hole formation, LasH ECHa and LasO ECHa are proposed to be inactive (40, 41) (Data Set S2D and E). An endo-β-methyl (α,β-unsaturated β-methyl) is predicted to form on module 10. The presence of a truncated ECH domain just upstream of an ECH2 domain has been commonly observed with the formation of exo-β-methylene (β,γ-unsaturated β-methylene), but to our knowledge, this is the first time such an architecture has been reported to form an endo-β-methyl (38).

10.1128/mbio.01524-22.4 DATA SET S2 (A) Alignment of *las* AT and AH domains with AT and AH domains from different *trans-*AT PKS pathways. Active sites as well as sites distinguishing AT and AH domains (M. Jenner, J. P. Afonso, C. Kohlhaas, P. Karbaum, S. Frank, J. Piel, N. J. Oldham, Chem Commun 52:5262–5265, 2016, https://doi.org/10.1039/C6CC01453D) have been marked. (B) Phylogenetic tree of AT and AH domains. The different types of domain separate into different clades (M. Jenner, J. P. Afonso, C. Kohlhaas, P. Karbaum, S. Frank, J. Piel, N. J. Oldham, Chem Commun 52:5262–5265, 2016, https://doi.org/10.1039/C6CC01453D). (C) Phylogenetic tree of ECH1 and ECH2 domains. Both the domains separate into different clades (S. T. Slocum, A. N. Lowell, A. Tripathi, V. V. Shende, J. L. Smith, D. H. Sherman, Methods Enzymol 604:207–236, 2018, https://doi.org/10.1016/bs.mie.2018.01.034). (D and E) Alignment of *las* ECH1 (D) and ECH2 (E) domains with respective ECH domains from other PKS pathways. Sequence that is required for the formation of the oxyanion hole, which stabilizes the enolate anions, is marked. LasH_a and LasO_a are proposed to be inactive, as they are truncated and show poor homology to the rest of the ECH domains (L. Gu, J. Jia, H. Liu, K. Håkansson, W. H. Gerwick, D. H. Sherman, J Am Chem Soc 128:9014–9015, 2006, https://doi.org/10.1021/ja0626382; M. A. Matilla, H. Stöckmann, F. J. Leeper, G. P. C. Salmond, J Biol Chem 287:39125–39138, 2012, https://doi.org/10.1074/jbc.M112.401026). (F) Alignment of *las* KS domains with active site residues (CHH) marked. “i” and “c” in the LasD KS indicate the incomplete and complete KS domain in different repeats of LasD, respectively. LasD is a decarboxylating KS; they are known to lack the active site cysteine (P. D. Walker, A. N. M. Weir, C. L. Willis, M. P. Crump, Nat Prod Rep 38:723–756, 2021, https://doi.org/10.1039/D0NP00045K). (G) Alignment of *las* KR domains with two from the erythromycin BGC to allow comparison. Active site residues and conserved motifs are marked. The presence or absence of the second aspartate in the LDD motif is supposed to predict the stereochemistry of the hydroxyl group (A. T. Keatinge-Clay, Chem Biol 14:898–908, 2007, https://doi.org/10.1016/j.chembiol.2007.07.009; P. Caffrey, ChemBioChem 4:654–657, 2003, https://doi.org/10.1002/cbic.200300581). Figures have been truncated for clarity and to show only the relevant sites. In phylogenetic trees, *las* BGC domains are highlighted in white. Download Data Set S2, PDF file, 8.0 MB. Copyright © 2022 Uppal et al. 2022 Uppal et al. https://creativecommons.org/licenses/by/4.0/ This content is distributed under the terms of the Creative Commons Attribution 4.0 International license.

Previously reported *trans*-AT PKS pathways featuring a monooxygenases (MOX) carrying out Baeyer-Villager (BV) oxidations, such as oocydin and sesbanimide (42–44) have done so in the context of a split module with an inactive dehydratase (DH) in the form KS-DH|MOX|ACP-KS. Therefore we propose that module 2 carries out a BV oxidation with the help of LasI. We also predict that the LasK monooxygenase installs an α-hydroxyl before loading onto module 3. According to a recent study on the oocydin pathway, hydroxylating monooxygenases are generally followed by an inactive KS domain (KS0), where the KS0 domain is essential for the hydroxylating function of the monooxygenase (44). This domain architecture has been identified in a number of different *trans*-AT PKS pathways present in diverse systems, including symbiont metagenomes (e.g., pederin biosynthesis [24]), free-living bacteria (e.g., labrenzin biosynthesis [45]), as well as free-living cyanobacteria (e.g., cusperin biosynthesis [46]). Similar domain architecture was identified in the *las* BGC, where LasK is followed by LasL KS0. We were unable to determine the stereochemistry of the inserted hydroxyl group, as this module architecture has previously been known to insert hydroxyl groups in both configurations. For example, hydroxyl groups inserted in oocydin (44), mupirocin, and thiomarinol (24) have configurations analogous to the 2-methyl groups controlled by 1-type ketoreductases (KRs) (47–49); in other words, 2*R* if the priority of C-1 is >C-3 and 2*S* if C-3 is >C-1, while the ones inserted in cusperin (46), nosperin (50), pederin (24), onnamide (51), and labrenzin (45) have the opposite configuration (analogous to 2-methyl groups controlled by 2-type KRs). Based on the recent reports that the most common transformation by cytochrome P450 enzymes in PKS biosynthesis is C-H hydroxylation (52), we suggest LasP to be oxidizing C-31. Another accessory protein, the enoylreductase (ER) domain LasQ (53), is proposed to be acting in *trans* as observed in other pathways, including lagriamide (54), patellazoles (55), and bacillaene (24, 56).

Due to the disruption of the catalytically active residues (CHH; Data Set S2F), we predict certain KS domains to be inactive (LasL KS1, LasL KS4, LasM KS5, and LasO KS7). We propose that the ACP domain of LasL directly takes the molecule from the first ACP of LasJ, and thus, we predict the KS domain in LasJ to be catalytically inactive despite the presence of catalytic residues, as observed in lagriamide, lankacidin, and etnangien pathways (24, 36). Likewise, the alignment of ketoreductase (KR) domains (Data Set S2G) allowed us to identify the ones lacking the KSY catalytic triad and thus identify the inactive KR domain in module 2 (LasL KR1). Additionally, it was found that the predicted stereoconfiguration of KR products (47, 48) in the *las* BGC matched the configuration of the equivalent moieties within the LSA structure produced by total synthesis (8). The absence of a KR domain required in module 14 is proposed to be compensated by a *trans*-acting KR likely from the following module as proposed in the patellazole (55) pathway.

We were able to identify two pyran synthase (PS) domains (in module 7 and module 13) based on their phylogeny (Data Set S3A) and alignment (57, 58) (Data Set S3B). These PS domains are at the correct position in the *las* BGC to insert the tetrahydropyran rings required to synthesize LSA. Even though module 13 lacks a DH domain required for pyran ring formation, we predict this role to be played by a *trans*-acting DH domain as commonly seen in *trans*-AT PKS pathways (24). As shown by Wagner et al., pyran synthase domains can catalyze the attack of either the *si* or the *re* face of the alkene (58). However, it was not possible to determine any sequence motif that could predict which face will be attacked (58), and as a result, we were unable to determine the stereochemical configuration of the tetrahydropyran ring. We were able to identify double bond-shifting DH domains in modules 4 (LasM DH1) and 8 (LasN DH1) by the absence of both proline in the HxxxGxxxxP motif and glutamine/histamine in the DxxxQ/H motif (Data Set S3C) (59). Moreover, alignment of the DH domains allowed us to identify the presence of inactive DH domains in modules 2 (LasH DH1) and 6 (LasM DH2) by the absence of both the catalytic histidine in the HxxxGxxxxP motif and the catalytic aspartic acid in the DxxxQ/H motif (Data Set S3D). LasL DH3 contains both the catalytic histidine in the HxxxGxxxxP and aspartic acid in DxxxQ/H motif, but it substitutes the proline in the HxxxGxxxxP motif with serine. Alignment of different DH domains with serine in the HxxxGxxxxP motif revealed a mixture of domains annotated as active and inactive (Data Set S3E). The majority of times, when the DH domain had the conserved histidine in the HxxxGxxxxP motif, it was annotated as active, and based on this, we propose LasL DH3 to be active.

10.1128/mbio.01524-22.5 DATA SET S3 (A) Phylogenetic tree of DH and PS domains, which separate into different clades (D. T. Wagner, Z. Zhang, R. A. Meoded, A. J. Cepeda, J. Piel, A. T. Keatinge-Clay, ACS Chem Biol 13:975–983, 2018, https://doi.org/10.1021/acschembio.8b00049). *Las* BGC DH/PS domains are highlighted in white. (B) Alignment of PS domains identified in the *las* BGC with PS domains from other *trans*-AT PKS pathways. The DH domain from the erythromycin BGC is used for comparison. LasO DH2 and LasM DH4 are annotated as putative PS domains. Generally, PS domains have an Hx_4_P motif instead of an Hx_8_P, and they lack the catalytic aspartate at the DxxxQ/H motif (D. T. Wagner, Z. Zhang, R. A. Meoded, A. J. Cepeda, J. Piel, A. T. Keatinge-Clay, ACS Chem Biol 13:975–983, 2018, https://doi.org/10.1021/acschembio.8b00049; P. Pöplau, S. Frank, B. I. Morinaka, J. Piel, Angew Chem Int Ed Engl 52:13215–13218, 2013, https://doi.org/10.1002/anie.201307406). This was found to be true only for LasM DH4 and not LasO DH2. However, identical variations from a traditional PS domain architecture are also seen in PS domains found in the mandelalide pathway (MndC DH3 and MndD DH3) (J. Lopera, I. J. Miller, K. L. McPhail, J. C. Kwan, mSystems 2:e00096–17, 2017, https://doi.org/10.1128/mSystems.00096-17). (C) Alignment of double bond-shifting DH domains identified in *las* BGC with similar domains found in other *trans*-AT PKS pathways. The DH domain from the erythromycin BGC is used for comparison. LasM DH1 and LasN DH1 are annotated as putative double bond-shifting DH domains. Generally, in DH-shifting domains, the conserved proline (P) in the Hx_8_P motif is often replaced by either valine (V) or leucine (L). In the case of LasM DH1, a methionine (M) instead of V or L appears in the place of P, which is in line with DH sequences observed in the difficidin BGC (X.-H. Chen, J. Vater, J. Piel, P. Franke, R. Scholz, K. Schneider, A. Koumoutsi, G. Hitzeroth, N. Grammel, A. W. Strittmatter, G. Gottschalk, R. D. Süssmuth, R. Borriss, J Bacteriol 188:4024–4036, 2006, https://doi.org/10.1128/JB.00052-06). Furthermore, DH-shifting domains are sometimes characterized by the replacement of the conserved aspartic acid (D) with asparagine (N) and substitution of glutamine (Q) or histidine (H) with V or L in the DxxxQ/H motif. Even though LasN DH1 has an N in place of D in the DxxxQ/H motif, it substitutes Q/H with a serine (S). This is unusual and not found in any other double bond-shifting DH. (D) Alignment of DH domains present in the *las* BGC with the DH domain from the erythromycin BGC. Putative PS and double bond-shifting DH domains have been excluded. LasH DH1 and LasM DH2 are annotated as inactive domains due to disrupted catalytic motifs Hx_8_P and DxxxQ/H. Even though in LasL DH1, the catalytic aspartic acid is replaced by glutamic acid (DxxxQ/H motif), we propose it is active, as a similar mutation is observed in the palmerolide BGC (N. E. Avalon, A. E. Murray, H. E. Daligault, C.-C. Lo, K. W. Davenport, A. E. K. Dichosa, P. S. G. Chain, B. J. Baker, Front Chem 9:802574, 2021, https://doi.org/10.3389/fchem.2021.802574). (E) Alignment of LasL DH3 with other DH domains having a serine in place of proline in the Hx_8_P motif. The DH domain from the erythromycin BGC is used for comparison. Sequence headers in blue represent DH domains annotated as active, while those in red are annotated as inactive. Download Data Set S3, PDF file, 4.1 MB. Copyright © 2022 Uppal et al. 2022 Uppal et al. https://creativecommons.org/licenses/by/4.0/ This content is distributed under the terms of the Creative Commons Attribution 4.0 International license.

**FIG 4:**
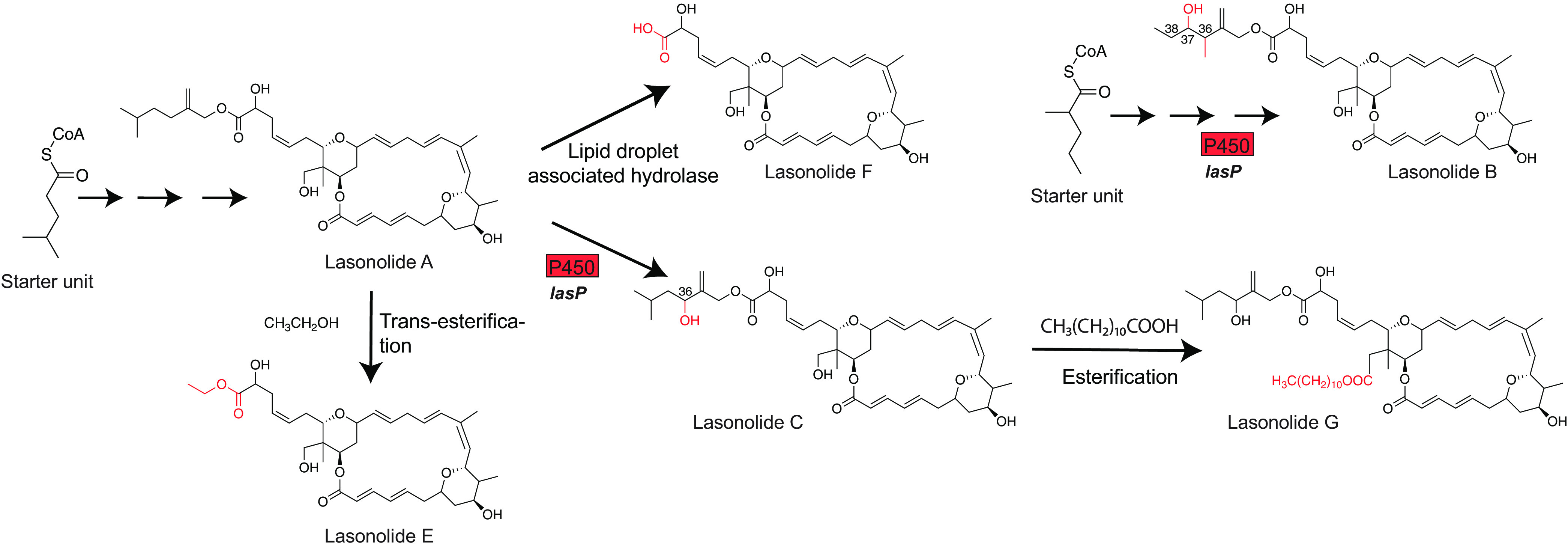
Proposed biosynthesis of lasonolide A analogs.

For the biosynthesis of other LSA analogs, we propose that lasonolide B results from an alternate starter unit, and all of them except for lasonolide D are modified post-PKS (Fig. 4). The cytochrome P450 LasP is predicted to oxidize LSA at C-36, leading to the synthesis of lasonolide C. Recently, it was shown that the serine hydrolase activity of lipid droplet-associated hydrolase is responsible for cleaving the ester bond in LSA, yielding the active form of the molecule, i.e., lasonolide F (60). Due to its hydrophobicity, LSA is able to easily diffuse into the plasma membrane and into lipid droplets, where it is converted into lasonolide F, a more hydrophilic molecule better able to diffuse out of the lipid droplet and into the cytoplasm to exhibit its cytotoxic effect (60). Lasonolide C seems to undergo an esterification reaction with a long-chain fatty acid [CH_3_(CH_2_)_10_COOH] to produce lasonolide G. We suggest that lasonolide E is biosynthesized by a *trans*-esterification reaction by reacting with an ethanol molecule. We suggest that the biosynthesis of lasonolide D is similar to that of LSA except that it starts with acetate as the starter unit loaded onto the ACP of LasJ, with LasH and LasI being inactive.

### Multiple repeats of the *las* BGC.

**FIG 5:**
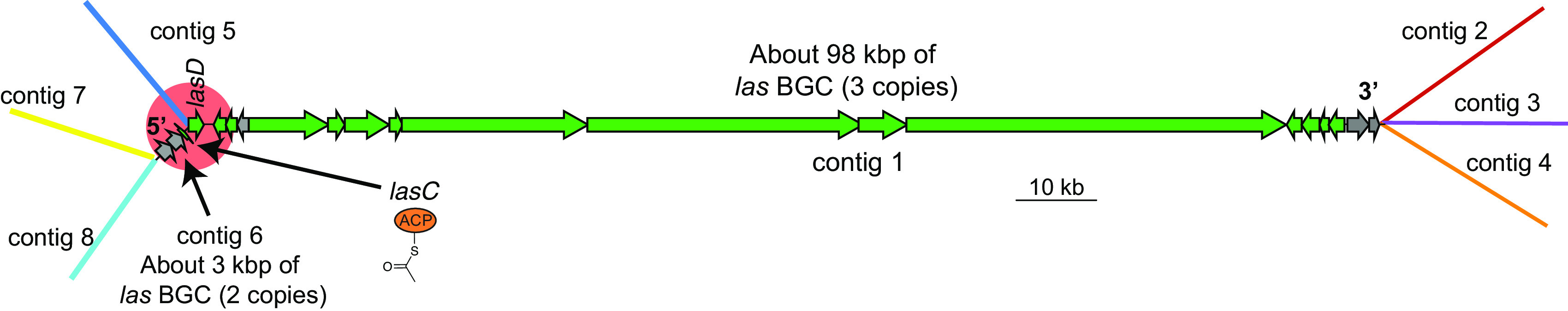
Model for three repeats of the *las* BGC. The 5′ end of *las* BGC is highlighted to demonstrate the location where one of the *las* BGC repeats lacks *lasC*. Contig(s) making up the 98-kbp segment of *las* BGC (one in *las* BGC_v1 and six in *las* BGC_v2) have been collectively referred to as contig 1. Contigs represented without gene arrows are not shown to scale.

The k-mer coverage of the *las* BGC (400.165× for *las* BGC_v1 and 159.02× for *las* BGC_v2) is roughly three times that of “*Ca.* Thermopylae lasonolidus” (135.16× in Forcepia_v1 and 48.24× in Forcepia_v2). The 3× coverage suggests three repeats of the putative *las* BGC. Visual inspection of the assembly graph, as well as mapping of the paired-end reads onto “*Ca.* Thermopylae lasonolidus,” allowed us to identify three connections on the 3′ end of *las* BGC but only two connections on the 5′ end of the pathway (contigs 7 and 8) (Fig. 5 and Table S3). Another contig (contig 5) was observed to be connected to *las* BGC about 3 kbp (3.6 kbp for *las* BGC_v1 and 3.7 kbp for *las* BGC_v2) from the 5′ end of *las* BGC. This suggests that the majority of *las* BGC (about 98 kbp) is repeated thrice, with a 3-kbp segment of the pathway (contig 6) being repeated twice (Fig. 5). The two repeats of contig 6 were further verified by more than twice the coverage of paired-end reads mapping to it compared to contig 5 (61), as well as its 2× coverage compared to the “*Ca.* Thermopylae lasonolidus” genome as a whole. All the connections between the *las* BGC and the bacterial genome were verified using PCR and Sanger sequencing of the amplicons. We believe that the three repeats of the *las* BGC might contribute to increased expression of LSA through increased gene dosage (62).

10.1128/mbio.01524-22.8 TABLE S3 Contigs making up the three repeats of the *las* BGC in “*Ca*. Thermopylae lasonolidus” in Forcepia_v1 and Forcepia_v2. Contig IDs represent the labels in Fig. 5 of the main text. Download Table S3, XLSX file, 0.07 MB. Copyright © 2022 Uppal et al. 2022 Uppal et al. https://creativecommons.org/licenses/by/4.0/ This content is distributed under the terms of the Creative Commons Attribution 4.0 International license.

**FIG 6:**
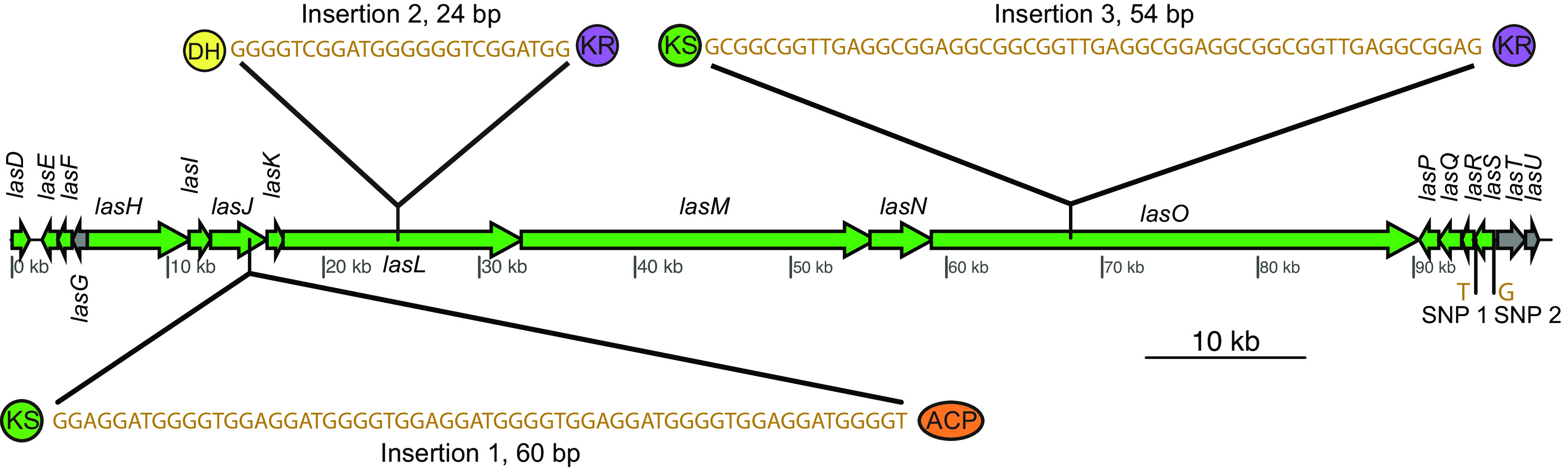
Variants identified between the three repeats of contig bin5_1_edit_8 (contig that makes up about 98 kbp of *las* BGC_v1).

On comparing the three repeats, it was observed that the *las* BGC repeat connected to contig 5 lacks *lasC* (ACP domain; highlighted area in Fig. 5), which is predicted to play an important role in β-branch formation. Furthermore, the same repeat which lacks *lasC* also shows the presence of an incomplete *lasD* (decarboxylating KS domain used in β-branching). Although this KS domain has the catalytic active site residues SHH, characteristic of decarboxylating KSs (38), it lacks about 47 amino acids that are present in the KS domain of the other two repeats connected to contig 6. On further investigation with GATK HaplotypeCaller (63, 64), we were able to detect three insertions and two single-nucleotide polymorphisms (SNPs) between the three repeats of contig gnl|UoN|bin5_1_edit_8 (the contig that makes up about 98 kbp of *las* BGC_v1) (Fig. 6 and Table 1). This was further supported by the allelic depth (AD) - informative reads supporting each allele - and Phred-scaled likelihoods (PL) of the possible genotypes. The genotype quality (GQ), which represents the confidence in the PL values, was 99 for all five variants, which is the maximum value GATK reports for GQ. Furthermore, alignment of *las* BGC_v1 with *las* BGC_v2 revealed that *las* BGC_v2 contains all the variants that were called by GATK, thus further supporting their presence. All three insertions are multiples of three base pairs (60 bp, 24 bp, and 54 bp) and thus do not cause any frameshift mutations. Moreover, all three insertions lie between domains within *trans-*AT PKS proteins, suggesting they do not contribute to functional differences. Change in one base from C to T at 93,995 bp does not result in a change in the amino acid sequence, as both codons (TAC and TAT) encode tyrosine. Finally, a change in the base from A to G at 95,154 bp lies just outside *lasS*, i.e., in the noncoding region. The above-mentioned differences in the three repeats of *las* BGC indicate that the repeats have been present long enough to allow divergence. However, the differences between them are not predicted to affect the function of the *las* BGC.

**TABLE 1:** Description of the variants identified between the three repeats of contig bin5_1_edit_8 (the contig that makes up about 98 kbp of *las* BGC_v1)^*a*^

ID	Location (bp)	Change	Length (bp)	Allelic depth	PL
Insertion 1	Between 15,246 and 15,247	+GGAGGATGGGGTGGAGGATGGGGTGGAGGATGGGGTGGAGGATGGGGTGGAGGATGGGGT	60	7, 15	1,129, 0
Insertion 2	Between 24,776 and 24,777	+GGGGTCGGATGGGGGGTCGGATGG	24	8, 57	3,092, 0
Insertion 3	Between 67,976 and 67,977	+GCGGCGGTTGAGGCGGAGGCGGCGGTTGAGGCGGAGGCGGCGGTTGAGGCGGAG	54	23, 164	5,987, 0
SNP 1	93,995	C→T	1	363, 457	2,973, 0
SNP 2	95,154	A→G	1	175, 621	16,101, 0

a Both allelic depth (AD) and PL values are represented in the manner “reference, variant.” A lower PL value represents a higher likelihood of the sample being that genotype.

### Evidence for horizontal gene transfer.

**FIG 7:**
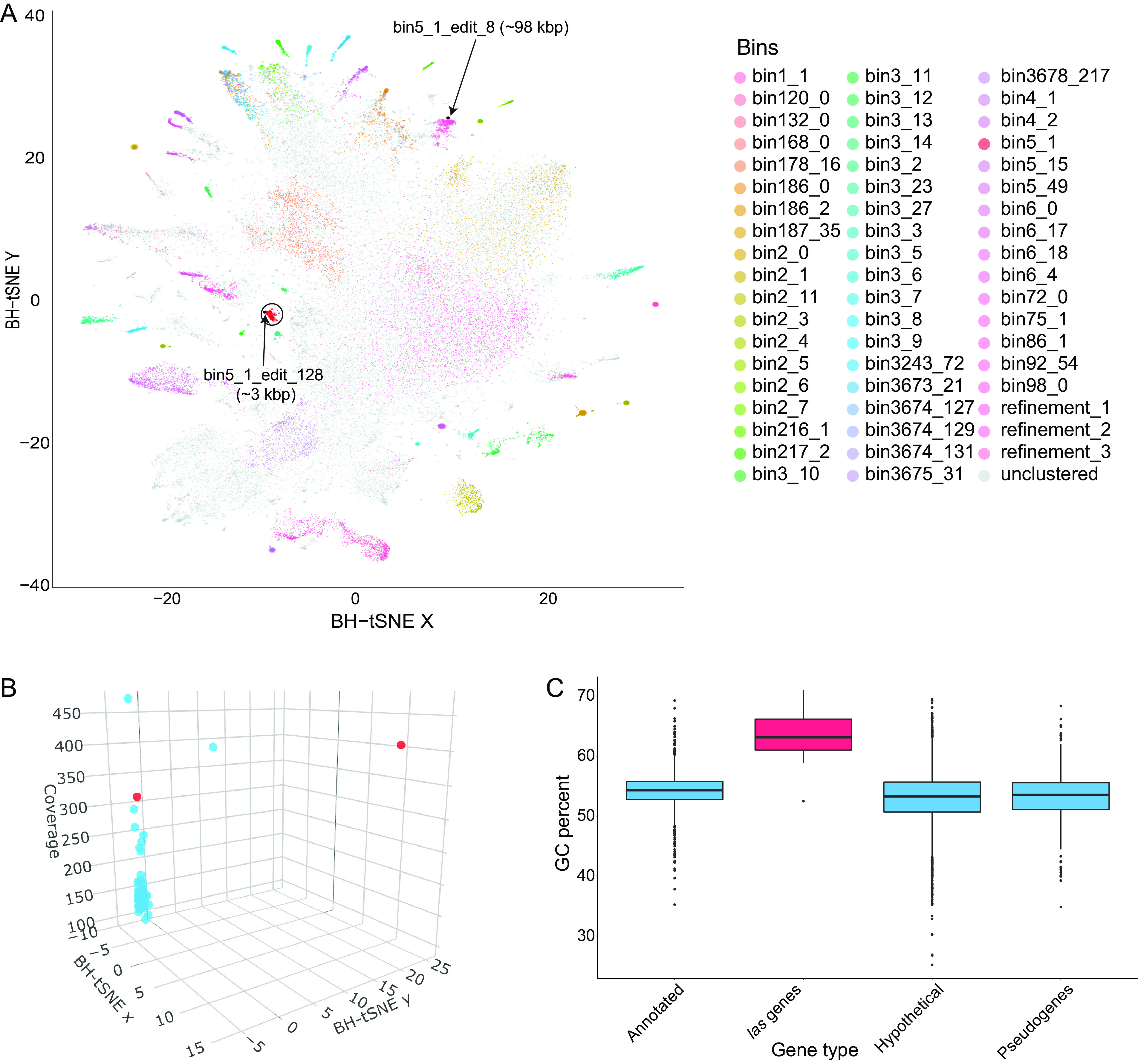
(A) Two-dimensional visualization of Autometa binning of Forcepia_v1. The “*Ca.* Thermopylae lasonolidus” genome is circled in black, and the *las* BGC contigs are marked with an arrow. Axes represent dimension-reduced Barnes-Hut stochastic neighbor embedding (BH-tSNE) values (BH-tSNE *x* and BH-tSNE *y*). (B) Three-dimensional visualization of contigs present in “*Ca.* Thermopylae lasonolidus.” The *las* BGC is colored red. Axes represent BH-tSNE values (BH-tSNE *x* and BH-tSNE *y*) along with k-mer coverage. (C) GC percentages of different sets of genes in Forcepia_v1 “*Ca.* Thermopylae lasonolidus.” The *las* BGC genes are colored red.

During the binning process by Autometa (65), Barnes-Hut stochastic neighbor embedding (BH-tSNE) was used to reduce 5-mer frequencies to two dimensions. Generally, contigs belonging to the same genome would have a similar 5-mer frequency and would be expected to cluster close to each other (66, 67). Visualization of the dimension-reduced data (Fig. 7A and B and Fig. S2A and B) revealed that the *las* BGC contigs significantly differ in their 5-mer frequency from “*Ca.* Thermopylae lasonolidus,” suggesting that the *las* BGC could have been recently horizontally acquired. Furthermore, the GC percentage of the *las* BGC is significantly different (*P* < 0.05, analysis of variance [ANOVA] followed by Tukey’s honestly significant difference [HSD]) from annotated, hypothetical, and pseudogenes (Fig. 7C and Fig. S2C), providing further evidence for horizontal transfer of the *las* BGC.

10.1128/mbio.01524-22.2 FIG S2 (A) Two-dimensional visualization of the initial Autometa binning of Forcepia_v2. The “*Ca.* Thermopylae lasonolidus” genome is circled in black, and contigs making up the *las* BGC are marked with arrows. Axes represent dimension-reduced Barnes-Hut stochastic neighbor-embedding (BH-tSNE) values (BH-tSNE *x* and BH-tSNE *y*). (B) Three-dimensional visualization of contigs present in the “*Ca.* Thermopylae lasonolidus” genome. Contigs making up the *las* BGC are colored red. (C) GC percentage of different sets of genes in Forcepia_v2 “*Ca.* Thermopylae lasonolidus.” *Las* BGC genes are colored in red. (D and E) Codon adaptation index (CAI) of different categories of genes present in Forcepia_v1 “*Ca.* Thermopylae lasonolidus” and Forcepia_v2 “*Ca.* Thermopylae lasonolidus,” respectively. *Las* BGC genes are colored in red. *P* values for pairwise comparison between different categories of genes are shown in the matrix below their respective plots. *P* values of <0.05 are considered significant. Other nonsignificant *P* values are colored red. Annotated and hypothetical genes represent the genes annotated with a function and genes annotated as hypothetical, respectively, by Prokka (T. Seemann, Bioinformatics 30:2068–2069, 2014, https://doi.org/10.1093/bioinformatics/btu153). Download FIG S2, PDF file, 1.9 MB. Copyright © 2022 Uppal et al. 2022 Uppal et al. https://creativecommons.org/licenses/by/4.0/ This content is distributed under the terms of the Creative Commons Attribution 4.0 International license.

The codon adaptation index (CAI) compares the synonymous codon usage of a gene and that of a reference set along with measuring the synonymous codon usage bias (68). The CAI for the *las* BGC was significantly different (*P* < 0.05, ANOVA followed by Tukey’s HSD) from the annotated, hypothetical, and pseudogenes, but it matched that of highly expressed genes (i.e., ribosomal proteins) (Fig. S2D and E). Thus, despite its horizontal acquisition, the BGC’s codon usage has been adapted to be efficiently translated even though the 5-mer composition is still different from the rest of the “*Ca.* Thermopylae lasonolidus” genome.

### The genome of the putative lasonolide A-producing symbiont.

“*Ca.* Thermopylae lasonolidus,” with multiple *las* BGC repeats, represents an important addition to the growing collection of symbiotic *Verrucomicrobia* (“*Candidatus* Didemnitutus mandela” and “*Candidatus* Synoicihabitans palmerolidicus”) being identified with repeated *trans*-AT PKS BGCs (62, 69, 70). Recently, two simultaneous studies have also identified a *trans*-AT PKS BGC for pateamine in a bacterium (“*Candidatus* Patea custodiens”) belonging to phylum *Kiritimatiellaeota* (71, 72), a recently proposed phylum which was previously classified within *Verrucomicrobia* (73). These findings highlight the importance of this understudied phylum as an important producer of natural products. “*Ca.* Thermopylae lasonolidus” is a little over 5 Mbp long and has a GC percentage of about 53%. It is estimated to be 99% complete, is 1.35% contaminated (74), and has tRNAs for all amino acids and complete 5S, 16S, and 23S rRNA genes. Based on MIMAG standards (25), the bin is classified as a high-quality MAG. Detailed statistics of the putative LSA producer are provided in Table 2.

**TABLE 2:** Genome statistics for “*Ca.* Thermopylae lasonolidus”^*a*^

Characteristic	Data for:	
	Forcepia_v1 “*Ca.* Thermopylae lasonolidus”	Forcepia_v2 “*Ca.* Thermopylae lasonolidus”
Size (Mbp)	4.85	4.93
Size (Mbp) after adding the three *las* repeats	5.05	5.13
checkM completeness (%)	99.24	99.32
checkM contamination (%)	1.35	1.35
No. of contigs	144	92
Longest contig (bp)	204,102	649,894
*N*_50_ (bp)	52,980	96,223
Avg GC%	53.81	53.88
% of pseudogenes out of total ORFs	16.31	16.62
No. of transposase genes	6	15
Coding density (%)^*a*^	79.45	79.41
Coding density without pseudogenes (%)^*a*^	72.58	72.38
Characteristics of eukaryotic-like proteins		
No. of ankyrin repeats	3	3
No. of tetratricopeptide repeat	43 (9 Sel-1 repeats)	42 (9 Sel-1 repeats)
No. of Pyrrolo-quinoline quinone-encoding genes	21	21
No. of leucine-rich repeats	16	16
No. of WD40 repeats	4	5

a Coding density is weighted by length, taking into account the 97.11% coding density of *las* BGC repeats.

Eukaryotic-like proteins (ELPs) are known to be present in genomes of sponge symbionts and have been found to play an important role in regulating their interaction with the host sponge (75–78). It is hypothesized that interaction with ELPs allows the symbiotic bacteria to evade phagocytosis by the sponge, thus allowing discrimination between food and symbiont bacteria (77, 79). A number of ELPs were identified in “*Ca.* Thermopylae lasonolidus” (Table 2 and Table S4A), thus suggesting a symbiotic relationship of the bacterium with *Forcepia* sp.

10.1128/mbio.01524-22.9 TABLE S4 Gene annotation in Forcepia_v1 and Forcepia_v2 “*Ca.* Thermopylae lasonolidus.” (A) Nonpseudogenes annotated as Eukaryotic-like proteins. (B) Genes forming the PV BMC cluster. (C) Nonpseudogenes annotated by dbCAN2. Download Table S4, XLSX file, 0.2 MB. Copyright © 2022 Uppal et al. 2022 Uppal et al. https://creativecommons.org/licenses/by/4.0/ This content is distributed under the terms of the Creative Commons Attribution 4.0 International license.

**FIG 8:**
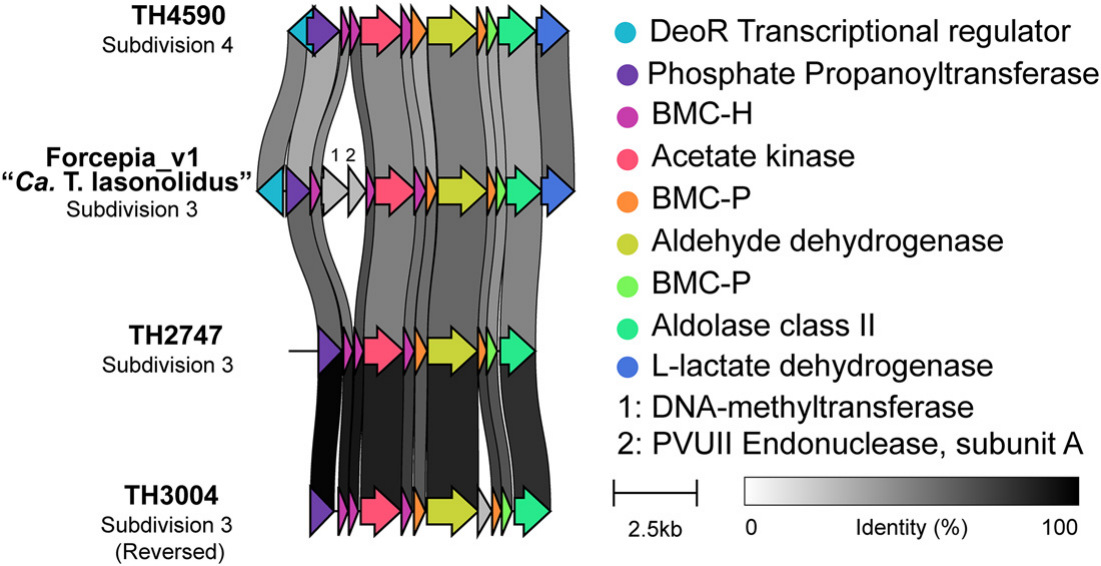
Comparison of the PV BMC gene cluster in Forcepia_v1 “*Ca.* Thermopylae lasonolidus” with the PV BMC clusters from other *Verrucomicrobia*. “*Ca.* Thermopylae lasonolidus” has DNA methyltransferase and PVUII endonuclease genes (in gray, labeled 1 and 2) between the first and the second BMC-H genes. This kind of arrangement was not observed in other PV BMC clusters.

Bacterial microcompartments (BMCs) are organelles that enclose enzymes within a selectively permeable proteinaceous shell (80), and they are rare among bacteria. Members of the phyla *Planctomycetes* and *Verrucomicrobia* have a unique BMC gene cluster called the *Planctomycetes*-*Verrucomicrobia* bacterial microcompartment (PV BMC), which is responsible for production of microcompartment shell proteins BMC-P and BMC-H as well as degradation of l-rhamnose, l-fucose, and fucoidans (76, 81, 82). Genes encoding the PV BMC cluster were identified in “*Ca.* Thermopylae lasonolidus” (Table S4B), and the respective gene clusters in Forcepia_v1 “*Ca.* Thermopylae lasonolidus” and Forcepia_v2 “*Ca.* Thermopylae lasonolidus” were found to be 100% identical using clinker (29). One interesting finding was that the identified PV BMC clusters had a DNA methyltransferase and a PVUII endonuclease gene between the first and the second BMC-H genes. This is different from the usual arrangement of the PV BMC gene cluster where both the BMC-H genes lie next to each other and the cluster lacks DNA-methyltransferase and PVUII endonuclease genes (Fig. 8). The presence of PV BMC genes in the “*Ca.* Thermopylae lasonolidus” genome suggests that it possesses bacterial microcompartments and that they might be involved in l-fucose and l-rhamnose degradation. Despite repeated attempts, we only found rhamnulokinase and fumarylacetoacetate hydrolase family proteins in the “*Ca*. Thermopylae lasonolidus” genomes, and we failed to identify other complementary enzymes involved in the degradation of l-fucose and l-rhamnose. However, other enzymes involved in carbohydrate metabolism, including glycoside hydrolases, carbohydrate binding module, polysaccharide lyase, carbohydrate esterases, and glycoside transferase, were detected (Table S4C), indicating that “*Ca.* Thermopylae lasonolidus” is capable of polysaccharide degradation, something that is observed in a number of marine *Verrucomicrobia* (83–85).

**FIG 9:**
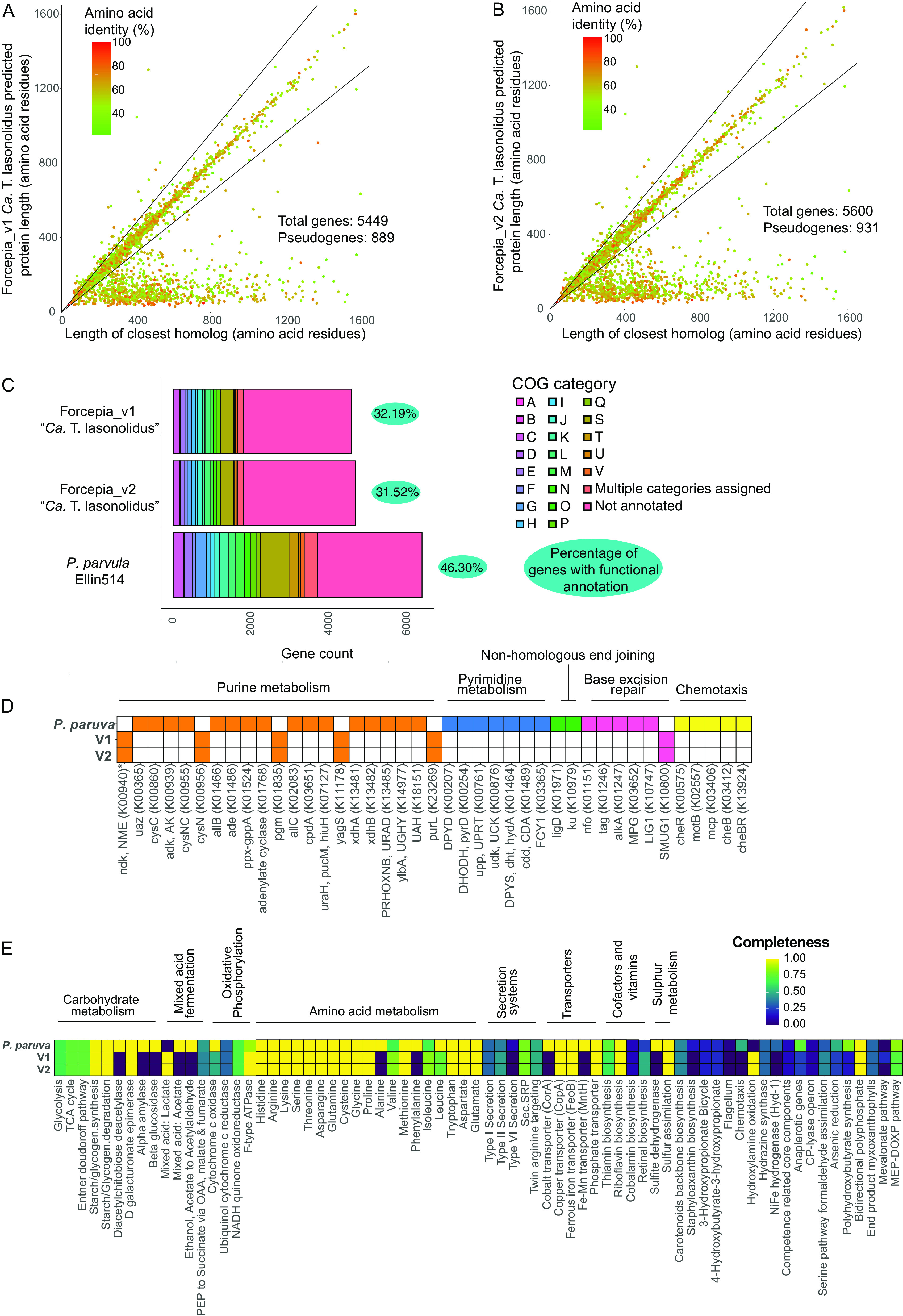
(A and B) Comparison of the gene length in Forcepia_v1 “*Ca.* Thermopylae lasonolidus” (A) and Forcepia_v2 “*Ca.* Thermopylae lasonolidus” (B), respectively, with their closest homologs in the nr database. Genes with length less than 80% of the closest homolog (below the lower black line) are classified as putative pseudogenes (62, 89, 90). The graphs have been truncated for clarity, as some predicted proteins have thousands of amino acid residues. (C) Comparison of functional COG categories in Forcepia_v1 “*Ca.* Thermopylae lasonolidus,” Forcepia_v2 “*Ca.* Thermopylae lasonolidus,” and P. paruva Ellin514 for nonpseudogenes. A gene is considered to have a functional annotation when it belongs to a COG category, except for category S, which represents unknown function. (D) Comparison of genes in different metabolic pathways for Forcepia_v1 “*Ca.* Thermopylae lasonolidus,” Forcepia_v2 “*Ca.* Thermopylae lasonolidus,” and P. paruva Ellin514, including only nonpseudogenes. Colored squares represent presence of a gene while white squares represent absence of gene. *K00940 is involved in both purine and pyrimidine metabolism. Genes absent in all three genomes have been removed. (E) Comparison of completeness of different metabolic pathways in Forcepia_v1 “*Ca.* Thermopylae lasonolidus,” Forcepia_v2 “*Ca.* Thermopylae lasonolidus,” and P. paruva Ellin514 (including only nonpseudogenes) as determined by KEGG decoder (108). Pathways have been grouped into categories wherever possible. Pathways absent in all three genomes have been removed. V1 and V2 refer to Forcepia_v1 “*Ca.* Thermopylae lasonolidus” and Forcepia_v2 “*Ca.* Thermopylae lasonolidus,” respectively.

A characteristic of obligate host-symbiont relationships is the loss of symbiont genes, which are required for independent survival. The early stages of genome reduction are characterized by reduced coding density and a high number of pseudogenes (86–88). We compared “*Ca* Thermopylae lasonolidus” with its closest free-living relative, Pedosphaera parvula Ellin514 (assembly accession no. GCA_000172555). The draft genome of P. paruva Ellin514 is 7.41 Mbp long, about 2.2 Mbp longer than “*Ca.* Thermopylae lasonolidus.” Furthermore, in P. paruva Ellin514 only 0.5% of total open reading frames (ORFs) were found to be pseudogenes (62, 89, 90) as opposed to about 16% in “*Ca.* Thermopylae lasonolidus” (Fig. 9A and B). Another indication of ongoing genome reduction is that a much smaller percentage of genes were annotated with putative functions in “*Ca.* Thermopylae lasonolidus” compared to P. paruva Ellin514 (Fig. 9C), perhaps indicating sequence degradation and divergence from functionally annotated genes. Moreover, compared with P. paruva Ellin514, “*Ca.* Thermopylae lasonolidus” lacks genes involved in DNA repair, DNA replication, chemotaxis, and nucleotide metabolism (Fig. 9D), a trend which is commonly observed in symbionts undergoing genome reduction (86). However, “*Ca.* Thermopylae lasonolidus” contains most of the primary metabolic pathways (Fig. 9E) compared to P. paruva Ellin514 and has a fairly large genome to be classified as reduced. Based on the above evidence, we suggest that “*Ca.* Thermopylae lasonolidus” is in early stages of genome reduction. This hypothesis is also supported by its low coding density of ~72% (without pseudogenes), relative to the average coding density of 85 to 90% for free-living bacteria (86), which suggests a recent transitional event, such as host restriction (86).

Due to its potency and unique mechanism of action, LSA is considered a potential anticancer drug lead; however, its limited supply has hampered its transition to clinical trials. The evidence provided here suggests that LSA is synthesized by a yet-uncultured verrucomicrobial symbiont, which harbors three copies of the putative *las* BGC. The detailed analysis of the biosynthetic scheme, genome characteristics of the putative producer, as well as the assembly of the *las* BGC on a plasmid will aid future cultivation and heterologous expression efforts.

## MATERIALS AND METHODS

For full details, see Text S1 in the supplemental material.

### Sponge collection.

*Forcepia* sp. (class, *Demospongiae*; order, *Poecilosclerida*; family, *Coelosphaeridae*) was collected in August of 2005 using the Harbor Branch Oceanographic Institute (HBOI) Johnson Sea Link submersible. Samples were collected at a depth of 70 m from the Gulf of Mexico (26.256573°N, 83.702772°W) on the Pulley Ridge (http://hboi-marine-biomedical-and-biotechnology-reference-collection.fau.edu/app/data-portal). The sponge samples were immediately frozen at −80°C. The sample ID was 12-VIII-05-1-006 200508121006 2005-08-12 JSL I-4837 (HBOI) *Forcepia* sp. strain 131921.

### DNA purification and sequencing.

The sponge hologenome was extracted using a modified cetyltrimethylammonium bromide (CTAB) DNA extraction method (51) and then size fractionated by low-melting-point gel electrophoresis. DNA fragments greater than 40 kb were recovered from the gel and used for fosmid library preparation (Text S1) as well as metagenomic sequencing. Two rounds of sequencing were performed for different DNA extracts from the *Forcepia* species sponge. For the first round (referred to as Forcepia_v1), Illumina TruSeq DNA libraries were prepared and sequenced by RTL Genomics using an Illumina MiSeq sequencer, giving us 108 million paired-end reads with length of 151 bp. For the second round of sequencing (referred to as Forcepia_v2), Illumina Nextera libraries were prepared and sequenced using a NovaSeq 6000 sequencer, giving us 303 million paired-end reads with length of 150 bp. Fosmids were sequenced by RTL Genomics and Genewiz.

### Identification and annotation of the *las* BGC.

Identification of the *las* BGC was done using tBLASTN (26), where KS domains from different *trans*-AT PKS pathways were used as a query against the metagenomic assembly (assembled using MetaSPAdes [91]; see Text S1). Genes for each bin were called and annotated using Prokka v1 (92, 93). MetaSPAdes contig headers have been replaced by their respective Prokka headers in the manuscript to maintain consistency with the annotation file submitted to NCBI. Genes on contigs making up the *las* BGC were not called correctly by Prokka (92, 93) and were thus annotated manually in Artemis (94) with the help of AntiSMASH (27), CDD (95), and SMART (96, 97).

### Functional analysis of the “*Ca.* Thermopylae lasonolidus” genome.

Genes called using Prokka v1 were used for the functional analysis (92, 93). PV BMC clusters were identified in “*Ca.* Thermopylae lasonolidus” using InterProScan v5.52-86.0 (98) and CDD (95). Initial identification of ELPs was done using Diamond BLASTP against the diamond-formatted nonredundant (nr) database (using parameters -k 1 --max-hsps 1) (99) and InterProScan v5.52-86.0 (98). This was followed by verification of nonpseudogenes using CDD (95). Enzymes involved in carbohydrate metabolism were detected using dbCAN2 (100) where genes annotated by ≥2 tools (out of HMMER, Diamond, and Hotpep) were kept. Clusters of orthologous groups (COG) categories were identified using the eggNOG-mapper online server (101, 102).

The genome of P. paruva Ellin514 was downloaded from GenBank (assembly accession no. GCA_000172555), and genes were called and annotated using Prokka v1 (92, 93). Primary metabolic pathways were identified for nonpseudogenes with kofamscan using the --mapper flag (103) and annotated against the KEGG database (104–106). The matrix with presence/absence of different enzymes was constructed in RStudio (107). Completeness of metabolic pathways was identified using KEGG-Decoder (108).

### Data availability.

The data associated with this study were deposited under BioProject accession no. PRJNA833117. The whole-genome sequencing (WGS) reads have been deposited in the Sequence Read Archive (SRA) with accession nos. SRR18966768 (Forcepia_v1) and SRR18966767 (Forcepia_v2). Sequences for bin5_1 and bin4_1 were deposited under the BioSample accession nos. SAMN27962571 and SAMN27962572, respectively. *Las* BGC v1 and v2 have been deposited to GenBank with accession numbers ON409579 and ON409580, respectively. *Las* BGC (*las* BGC_v1) have been submitted to MIBiG with accession no. BGC0002153.
