# Supplementary material for: Uncovering Lasonolide A Biosynthesis Using Genome-Resolved Metagenomics
Source: mBio. 2022 Sep 20;13(5):e01524-22. doi: 10.1128/mbio.01524-22 (PMC9600693; doi:10.1128/mbio.01524-22)
Supplement: TEXT S1 [file mbio.01524-22-s0004.docx]

Supplementary methods

## Metagenomic fosmid library construction

The sponge hologenome was extracted using a modified cetyltrimethylammonium bromide DNA extraction method [(1)](https://paperpile.com/c/hIwQud/r8F6Q). The crude metagenomic DNA was then size-fractionated by low melting point gel electrophoresis and DNA fragments greater than 40 kb were recovered from the gel. The purified DNA was used to construct a fosmid library using Epicentre’s CopyControl Cloning System following the manufacturer’s protocols [(2)](https://paperpile.com/c/hIwQud/CcDk0). Briefly, purified *Forcepia* metagenomic DNA was blunt-ended and then ligated into the Cloning-Ready CopyControl pCC2FOS Vector, packaged into Lambda Phage T1 and transformed into EPI300-T1R *E. coli* host cells. Based on the titer of the phage particles, the library contained approximately 600,000 clones. Transformed cells were mixed into semiliquid LB with 12.5 µg/mL chloramphenicol (as a selection marker) to produce ~1000 cfus/mL as previously described. This volume was then distributed as 1 mL aliquots into 631 2 mL screw cap tubes which were denoted as “pools” [(2, 3)](https://paperpile.com/c/hIwQud/fs6BP+CcDk0).

## Fosmid library screening before WGS

For metagenomic library screening before WGS, degenerate primers were designed to target conserved PKS genes including HMG-CoA synthase, free-standing KS and ACP, and two ECH domains (see **Table S1A**). For the PCR reaction, *Forcepia* metagenomic DNA was used as the template along with the GoTaq Green Master Mix (Promega). PCR conditions were as follows: initial denaturing at 95° C for 5 min; denaturing at 95° C for 30 sec, annealing (temperature specific for each primer: ~56° C) for 30s, and extension at 72° C for 1 min for a total of 35 cycles followed by a final extension at 72° C for 5 min. PCR products were cloned into the pGMET vector (Promega) and subjected to Sanger sequencing (Eurofins Genomics). Sequence analysis of PCR products including alignment and phylogenetic analysis were conducted to determine their potential involvement in biosynthesis of complex polyketides. The primers and PCR conditions described above were then used to screen the metagenomic library, consisting of 631 pools containing 1000 cfu per tube. To reduce the initial screening process, aliquots were taken from each pool in a row and added to a separate tube labeled as a superpool. Superpools were screened to detect putative *las* biosynthetic genes using 2 μL of the bacterial suspension as template (total volume: 25 μL). Superpools in which *las* genes were detected were then chosen for screening of the individual pools used to construct the superpool. Following identification of positive pools, single clones carrying putative *las* biosynthetic genes were isolated via multiple rounds of whole cell PCR and dilution of the semiliquid pools, a method which has been previously described [(3)](https://paperpile.com/c/hIwQud/fs6BP). Single colonies which were positive for *las* genes were then grown up to isolate fosmid DNA using the QIAprep Spin Miniprep Kit. Select fosmids were then sent for Illumina sequencing at RTL Genomics and Genewiz. Upon capture and sequencing of fosmid 6-71, a primer walking approach was utilized to capture approximately 48kb of the putative *las* BGC at its 3′ end (fosmids 5-16, 6-71, 3-46, 1-80, and 4-77) (**Fig. S1A**). However, minimal progress was made toward capturing the remaining half of the BGC as primer walking failed to produce new hits in the region upstream of fosmid 5-16.

## Fosmid library screening after WGS

## For library screening following WGS, primer sets were designed to amplify unique genes within the uncaptured regions of the BGC (**Table S1C**). PCR conditions were performed as previously described in the “fosmid library screening before WGS” section. The 70 superpools were screened using all newly designed primer sets to detect *las* biosynthetic genes. Gel electrophoresis was performed to visualize the results and all superpools were given scores based on band intensity for each primer set. Superpools which had strong signals for multiple, adjacent primer sets were chosen for further screening. Pools which corresponded to the selected superpools were screened individually with the same primer sets. Pools that had the strongest PCR signals for multiple adjacent primer sets were chosen for dilution and further screening until single clones were captured. Once single clones were identified, the fosmid DNA was isolated as before and used for a final multi-primer screening to determine the predicted pathway coverage of the DNA insert. PCR bands were extracted and sent for Sanger sequencing at Eurofins Genomics. Based on these results, select fosmids, 5-41, 2-18, and 2-13, were sent for Illumina sequencing at RTL Genomics.

## DNA Assembly and Validation of Transformants

DNA assembly of 5 Cas9-restricted fosmid preparations (5-41, 2-18, 2-13, 5-16 and 4-77) was performed into 2 linearized intermediate BAC vectors containing overlap regions specific to the target assembly, in order to produce a LEFT fragment containing 3 fosmid fragments (5-41, 2-18 and 2-13) and a RIGHT (5-16 and 4-77) fragment containing 2 fosmid fragments. The repetitive sequence of the LEFT fragment caused frequent mis-cutting or mis-assembly, resulting in a need to explore alternate designs. The LEFT and RIGHT fragments were validated by colony PCR, junction sequencing, and restriction digest mapping. The LEFT and RIGHT fragments were then restricted again and assembled into a linearized pDualP vector. The DNA assembly reaction was transformed into *E. coli* BacOpt2.0 cells from which colonies were recovered. Screening of over 100 colonies showed frequent rearrangement and loss of genetic material in the middle of the LEFT fragment area. Three colonies were recovered that passed validation by colony PCR at 7 regions spanning the BGC, junction sequencing, and restriction digest mapping. Clones for the construct were restriction-digested separately by EcoRI and BamHI and compared to a simulated digest, confirming a match. The junction PCRs for these clones were purified and Sanger sequenced to confirm the DNA assembly as designed. Primers used for DNA assembly and subsequent validation by PCR are found in **Table S1D**.

## Metagenomic binning, annotation and taxonomic classification

The sequenced paired-end reads were trimmed of adapters using Trimmomatic [(4)](https://paperpile.com/c/hIwQud/v3oKT) and then assembled using MetaSpades [(5)](https://paperpile.com/c/hIwQud/wLtYg). Metagenomic binning for both assemblies (Forcepia_v1 and Forcepia_v2) was performed using Autometa v1 [(6)](https://paperpile.com/c/hIwQud/oWgeb) with the --maketaxtable option, specifying a length cutoff of 500bp and 3000bp for Forcepia_v1 and Forcepia_v2, respectively. The bins were manually curated by taking into account the coverage and taxonomy of the contigs as well as using an interactive dashboard - Automappa (<https://github.com/WiscEvan/Automappa>).

## Two additional contigs were also added to Forcepia_v1: bin5_1 - gnl|UoN|bin5_1_edit_144 and gnl|UoN|bin5_1_edit_143. PCR, Sanger sequencing and the assembly graph were used to verify that they are present in bin5_1. Furthermore, three additional contigs (gnl|UoN|bin4_1_edit_3, gnl|UoN|bin4_1_edit_2 and gnl|UoN|bin4_1_edit_1) were added to Forcepia_v2 bin4_1 based on the assembly graph and mapping of paired-end reads.

## Binning metrics and taxonomic classification

Completeness and contamination of the metagenomic bins were calculated using CheckM v1.1.3 [(7)](https://paperpile.com/c/hIwQud/XDNyO), utilizing the lineage workflow, and taxonomies of metagenomic bins were predicted using GTDB-tk v1.5.0 (reference data version r202) [(8)](https://paperpile.com/c/hIwQud/k0DLZ) utilizing the classify workflow. It was observed that both CheckM and GTDB-tk were using translation table 4 for “*Ca* T. lasonolidus” even though the bin was placed under phylum Verrucomicrobiota which as per NCBI (<https://www.ncbi.nlm.nih.gov/Taxonomy/Utils/wprintgc.cgi>) should be using translation table 11. To make use of the correct translation table, genes externally called using Prokka v1 [(9)](https://paperpile.com/c/hIwQud/3WqlK) were provided to CheckM using the --genes flag. In the case of GTDB-tk a batch file was provided indicating the use of table 11.

## Confirmation of terminal connections to the *las* BGC

Paired-end reads were mapped using Bowtie2 [(10)](https://paperpile.com/c/hIwQud/qXDVi) on the MetaSpades assembly, using the --very-sensitive option. Calculation of the number of paired-end reads connecting contigs was done using the cytoscapeviz.pl script [(11)](https://paperpile.com/c/hIwQud/qHJgP) and then visualized in cytoscape [(12)](https://paperpile.com/c/hIwQud/qpU1U).

Primers found in **Table S1B** were designed to amplify the junctions between the contigs upstream and downstream of the *las* BGC depicted in **Fig. 5**. PCR was performed using the Phusion High-Fidelity PCR Kit and high molecular weight *Forcepia* DNA as template. PCR conditions were as follows: Initial denaturing at 98° C for 30 s, denaturing at 98° C for 10 s, annealing gradient for 30 s, and extension at 72° C for 30 min for a total of 40 cycles followed by a final extension at 72° C for 5 min. Sanger sequencing (Eurofins Genomics) of the amplicons and alignment to the putative connections with contig gnl|UoN|bin5_1_edit_8 were used to experimentally confirm the linkages between the contigs depicted in **Fig. 5**.

## Manual curation of the *las* BGC contig

Within contig gnl|UoN|bin5_1_edit_8, regions of short tandem repeats led to four sequence assembly gaps (48953-49052bp, 49095-49194bp, 68196-68295bp, and 95498-95597bp). To resolve these errors, trimmed reads were aligned to gnl|UoN|bin5_1_edit_8 using the Integrative genomics viewer [(13–15)](https://paperpile.com/c/hIwQud/p4AOR+txYwG+RlG2z). These reads were then manually inspected and re-assembled to fill in the assembly gaps. These results were then compared to the assemblies of the fosmid DNA which verified the manually assembled regions.

## Bioinformatic analysis

rRNA genes in the “*Ca*. T. lasonolidus” genome were identified using Barrnap v0.9 (<https://github.com/tseemann/barrnap>).

Domain sequences were aligned using Clustal Omega [(16)](https://paperpile.com/c/hIwQud/WHrB1). Alignments were manually inspected and trimmed prior to phylogenetic tree construction using FasttreeMP [(17)](https://paperpile.com/c/hIwQud/YwWkZ) with parameters -slow -spr 10 -mlacc 3 -bionj -gamma. The tree was visualized with the Interactive Tree of Life server [(18)](https://paperpile.com/c/hIwQud/NykoU).

Variant calling was done using GATK HaplotypeCaller following best practices as previously described [(19, 20)](https://paperpile.com/c/hIwQud/THf4e+l3Fjh).

The phylogenetic tree of 51 different Verrucomicrobial genomes was constructed using Phylophlan3 v3.0.60 [(21)](https://paperpile.com/c/hIwQud/zXF3V). Bootstrap values were calculated using RAxML [(22)](https://paperpile.com/c/hIwQud/7kBxl) with 1000 bootstrap replicates, and the following parameters: -f a -m PROTGAMMAAUTO -# 1000 -p 1989 -x 1989.

Coding density was calculated as a percentage of coding sequence upon total sequence count. In case of gene overlap the overlap region was only counted once.

## Identification of pseudogenes

Diamond blastp [(23)](https://paperpile.com/c/hIwQud/4Zjt8) was performed for genes called using Prokka against the diamond-formatted nr database with -k 1 --max-hsps 1 options. Genes that were more than 20% shorter than their respective best BLASTP hits in the nr database were annotated as pseudogenes as described previously [(24–26)](https://paperpile.com/c/hIwQud/ENIJ2+A00at+syTvu). *Las* genes are assumed to be non-pseudogenes.

## CAI calculation

CAI was calculated using the formula of Sharp and Li [(27)](https://paperpile.com/c/hIwQud/yec06), and ribosomal proteins were used to calculate the *w* value and relative synonymous codon usage. Genes annotated with functions by Prokka [(9)](https://paperpile.com/c/hIwQud/3WqlK) were categorized as annotated, while genes not annotated were categorized as hypothetical. Ribosomal proteins were identified using Prokka [(9)](https://paperpile.com/c/hIwQud/3WqlK) annotation. CAI plots were produced using the ggplot2 [(28)](https://paperpile.com/c/hIwQud/soUa9) package in R. The statistical significance of the differences between the groups was assessed using one-way analysis of variance (ANOVA) followed by Tukey’s honest significant difference (HSD) test using the aov and TukeyHSD function respectively in RStudio.

# **References for the supplemental methods**

1. [Piel J, Hui D, Wen G, Butzke D, Platzer M, Fusetani N, Matsunaga S. 2004. Antitumor polyketide biosynthesis by an uncultivated bacterial symbiont of the marine sponge *Theonella swinhoei*. Proc Natl Acad Sci U S A 101:16222–16227.](http://paperpile.com/b/hIwQud/r8F6Q)

2. [Gurgui C, Piel J. 2010. Metagenomic approaches to identify and isolate bioactive natural products from microbiota of marine sponges. Methods Mol Biol 668:247–264.](http://paperpile.com/b/hIwQud/CcDk0)

3. [Hrvatin S, Piel J. 2007. Rapid isolation of rare clones from highly complex DNA libraries by PCR analysis of liquid gel pools. J Microbiol Methods 68:434–436.](http://paperpile.com/b/hIwQud/fs6BP)

4. [Bolger AM, Lohse M, Usadel B. 2014. Trimmomatic: A flexible trimmer for Illumina sequence data. Bioinformatics 30:2114–2120.](http://paperpile.com/b/hIwQud/v3oKT)

5. [Nurk S, Meleshko D, Korobeynikov A, Pevzner PA. 2017. metaSPAdes: a new versatile metagenomic assembler. Genome Res 27:824–834.](http://paperpile.com/b/hIwQud/wLtYg)

6. [Miller IJ, Rees ER, Ross J, Miller I, Baxa J, Lopera J, Kerby RL, Rey FE, Kwan JC. 2019. Autometa: Automated extraction of microbial genomes from individual shotgun metagenomes. Nucleic Acids Res 47:e57.](http://paperpile.com/b/hIwQud/oWgeb)

7. [Parks DH, Imelfort M, Skennerton CT, Hugenholtz P, Tyson GW. 2015. CheckM: Assessing the quality of microbial genomes recovered from isolates, single cells, and metagenomes. Genome Res 25:1043–1055.](http://paperpile.com/b/hIwQud/XDNyO)

8. [Chaumeil P-A, Mussig AJ, Hugenholtz P, Parks DH. 2020. GTDB-Tk: A toolkit to classify genomes with the Genome Taxonomy Database. Bioinformatics 36:1925–1927.](http://paperpile.com/b/hIwQud/k0DLZ)

9. [Seemann T. 2014. Prokka: Rapid prokaryotic genome annotation. Bioinformatics 30:2068–2069.](http://paperpile.com/b/hIwQud/3WqlK)

10. [Langmead B, Salzberg SL. 2012. Fast gapped-read alignment with Bowtie 2. Nat Methods 9:357–359.](http://paperpile.com/b/hIwQud/qXDVi)

11. [Albertsen M, Hugenholtz P, Skarshewski A, Nielsen KL, Tyson GW, Nielsen PH. 2013. Genome sequences of rare, uncultured bacteria obtained by differential coverage binning of multiple metagenomes. Nat Biotechnol 31:533–538.](http://paperpile.com/b/hIwQud/qHJgP)

12. [Shannon P, Markiel A, Ozier O, Baliga NS, Wang JT, Ramage D, Amin N, Schwikowski B, Ideker T. 2003. Cytoscape: A software environment for integrated models of biomolecular interaction networks. Genome Res 13:2498–2504.](http://paperpile.com/b/hIwQud/qpU1U)

13. [Thorvaldsdóttir H, Robinson JT, Mesirov JP. 2013. Integrative Genomics Viewer (IGV): High-performance genomics data visualization and exploration. Brief Bioinformatics 14:178–192.](http://paperpile.com/b/hIwQud/p4AOR)

14. [Robinson JT, Thorvaldsdóttir H, Winckler W, Guttman M, Lander ES, Getz G, Mesirov JP. 2011. Integrative genomics viewer. Nat Biotechnol 29:24–26.](http://paperpile.com/b/hIwQud/txYwG)

15. [Robinson JT, Thorvaldsdóttir H, Wenger AM, Zehir A, Mesirov JP. 2017. Variant review with the integrative genomics viewer. Cancer Res 77:e31–e34.](http://paperpile.com/b/hIwQud/RlG2z)

16. [Sievers F, Wilm A, Dineen D, Gibson TJ, Karplus K, Li W, Lopez R, McWilliam H, Remmert M, Söding J, Thompson JD, Higgins DG. 2011. Fast, scalable generation of high-quality protein multiple sequence alignments using Clustal Omega. Mol Syst Biol 7:539.](http://paperpile.com/b/hIwQud/WHrB1)

17. [Price MN, Dehal PS, Arkin AP. 2010. FastTree 2 – Approximately maximum-likelihood trees for large alignments. PLoS One 5:e9490.](http://paperpile.com/b/hIwQud/YwWkZ)

18. [Letunic I, Bork P. 2019. Interactive Tree Of Life (iTOL) v4: Recent updates and new developments. Nucleic Acids Res 47:W256–W259.](http://paperpile.com/b/hIwQud/NykoU)

19. [Van der Auwera GA, O’Connor BD. 2020. Genomics in the Cloud: Using Docker, GATK, and WDL in Terra. O’Reilly Media, Inc.](http://paperpile.com/b/hIwQud/THf4e)

20. [DePristo MA, Banks E, Poplin R, Garimella KV, Maguire JR, Hartl C, Philippakis AA, del Angel G, Rivas MA, Hanna M, McKenna A, Fennell TJ, Kernytsky AM, Sivachenko AY, Cibulskis K, Gabriel SB, Altshuler D, Daly MJ. 2011. A framework for variation discovery and genotyping using next-generation DNA sequencing data. Nat Genet 43:491–498.](http://paperpile.com/b/hIwQud/l3Fjh)

21. [Asnicar F, Thomas AM, Beghini F, Mengoni C, Manara S, Manghi P, Zhu Q, Bolzan M, Cumbo F, May U, Sanders JG, Zolfo M, Kopylova E, Pasolli E, Knight R, Mirarab S, Huttenhower C, Segata N. 2020. Precise phylogenetic analysis of microbial isolates and genomes from metagenomes using PhyloPhlAn 3.0. Nat Commun 11:2500.](http://paperpile.com/b/hIwQud/zXF3V)

22. [Stamatakis A. 2014. RAxML version 8: A tool for phylogenetic analysis and post-analysis of large phylogenies. Bioinformatics 30:1312–1313.](http://paperpile.com/b/hIwQud/7kBxl)

23. [Buchfink B, Xie C, Huson DH. 2015. Fast and sensitive protein alignment using DIAMOND. Nat Methods 12:59–60.](http://paperpile.com/b/hIwQud/4Zjt8)

24. [Kwan JC, Schmidt EW. 2013. Bacterial endosymbiosis in a chordate host: Long-term co-evolution and conservation of secondary metabolism. PLoS ONE 8:e80822.](http://paperpile.com/b/hIwQud/ENIJ2)

25. [Lerat E, Ochman H. 2005. Recognizing the pseudogenes in bacterial genomes. Nucleic Acids Res 33:3125–3132.](http://paperpile.com/b/hIwQud/A00at)

26. [Waterworth SC, Flórez LV, Rees ER, Hertweck C, Kaltenpoth M, Kwan JC. 2020. Horizontal gene transfer to a defensive symbiont with a reduced genome in a multipartite beetle microbiome. mBio 11:e02430–19.](http://paperpile.com/b/hIwQud/syTvu)

27. [Sharp PM, Li W-H. 1987. The codon adaptation index-a measure of directional synonymous codon usage bias, and its potential applications. Nucleic Acids Res 15:1281–1295.](http://paperpile.com/b/hIwQud/yec06)

28. [Wickham H. 2016. ggplot2: Elegant Graphics for Data Analysis. Springer-Verlag New York.](http://paperpile.com/b/hIwQud/soUa9)
