# Supplementary figures and images for: Uncovering Lasonolide A Biosynthesis Using Genome-Resolved Metagenomics
Source: mBio. 2022 Sep 20;13(5):e01524-22. doi: 10.1128/mbio.01524-22 (PMC9600693; doi:10.1128/mbio.01524-22)

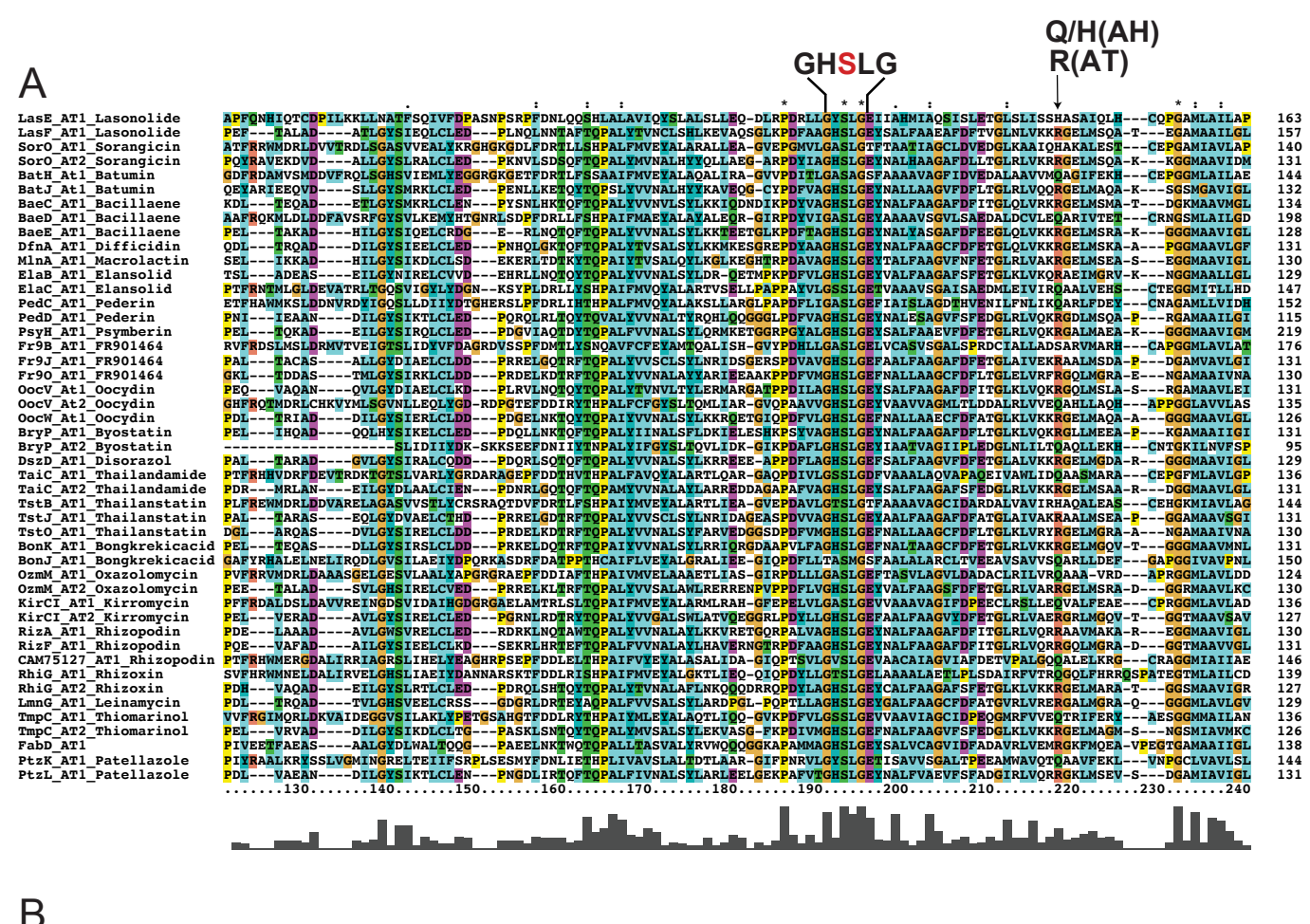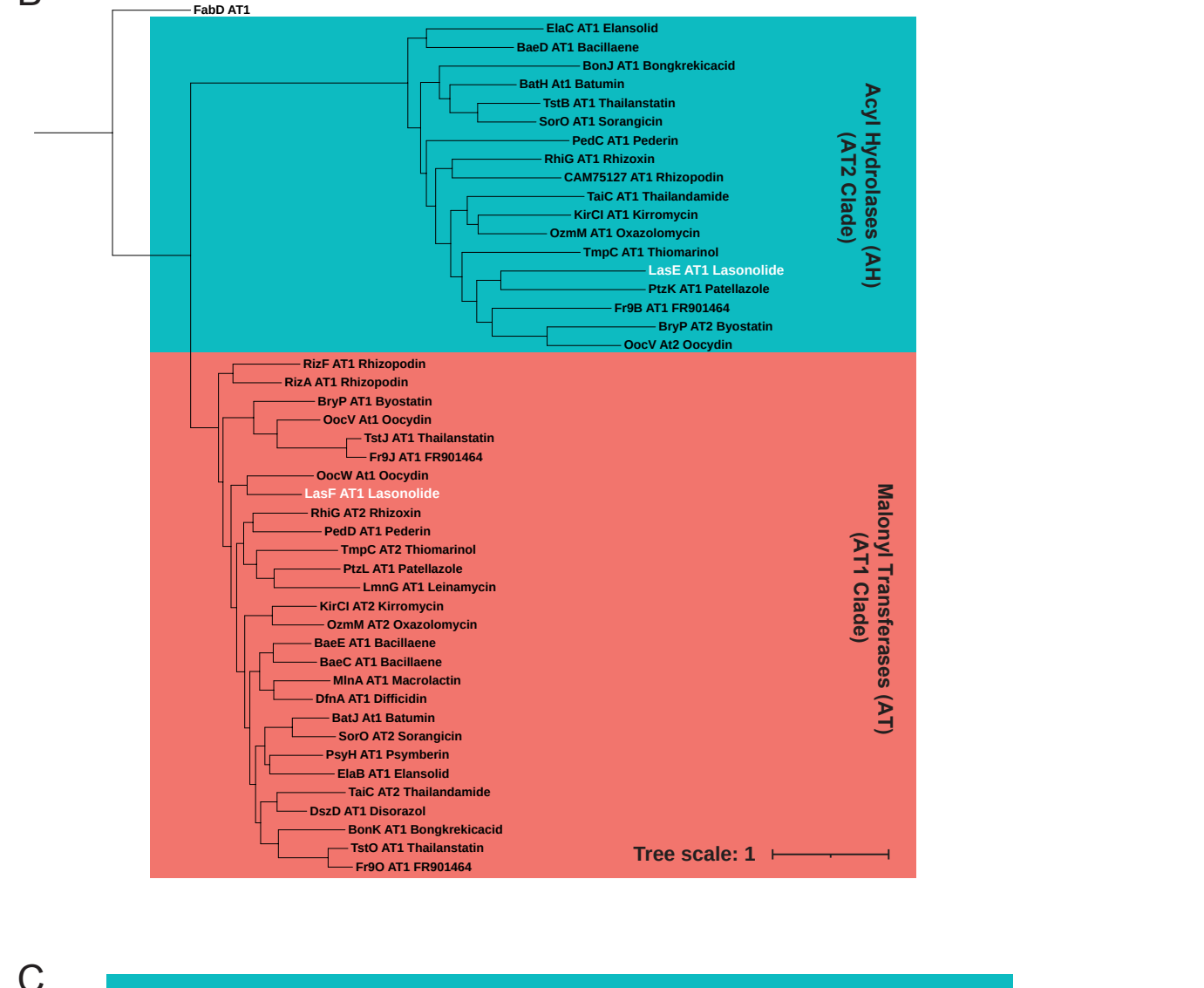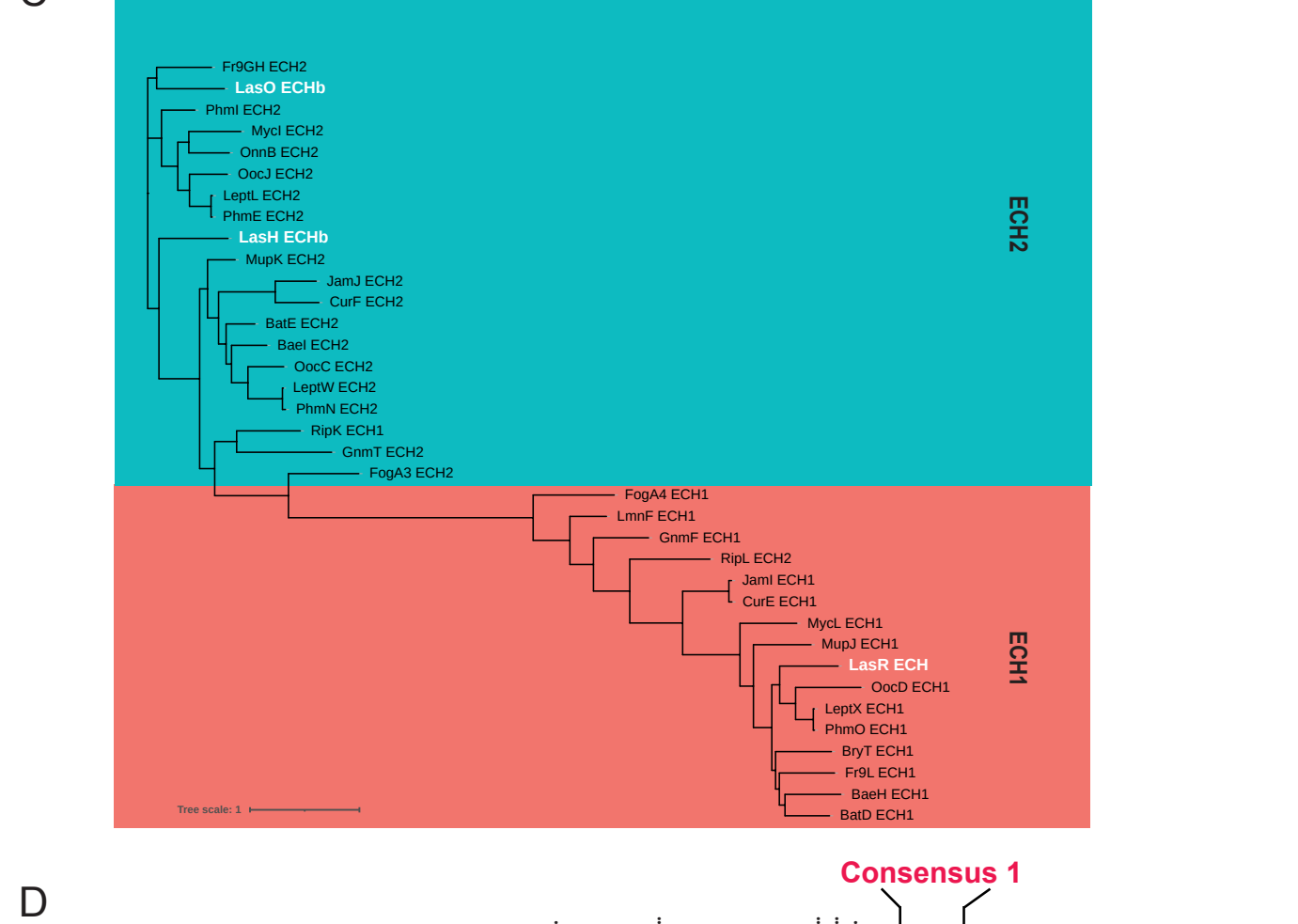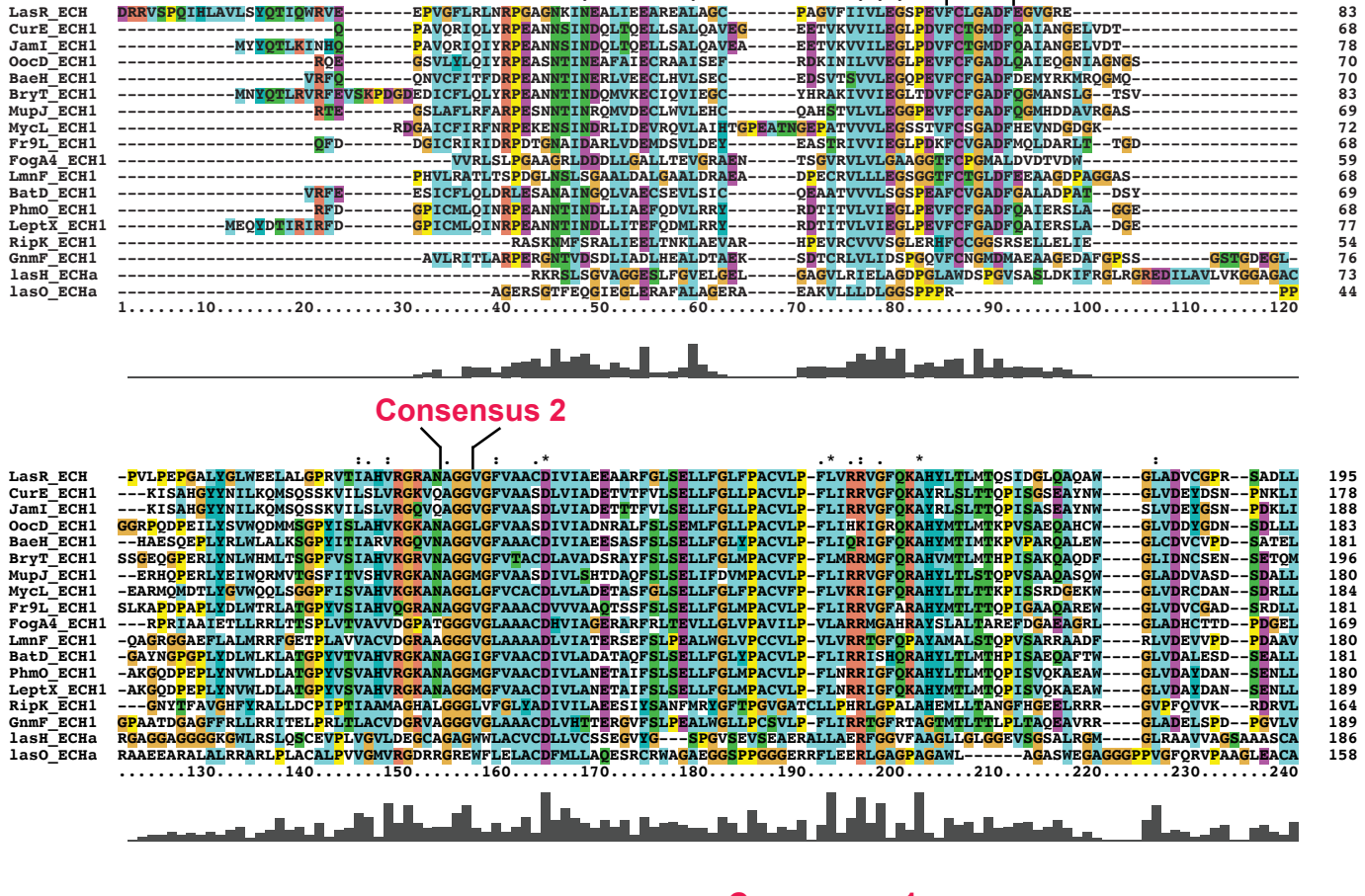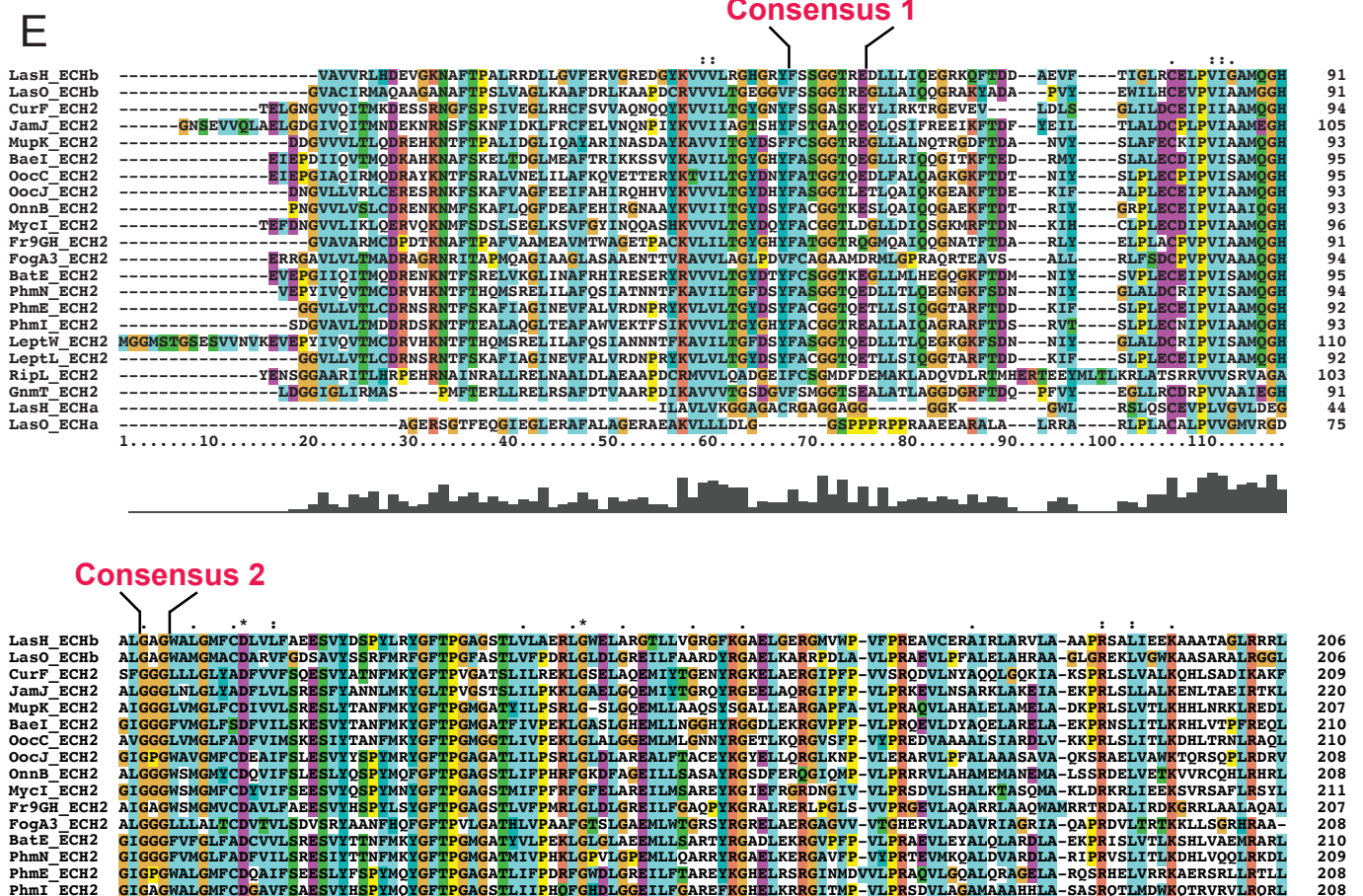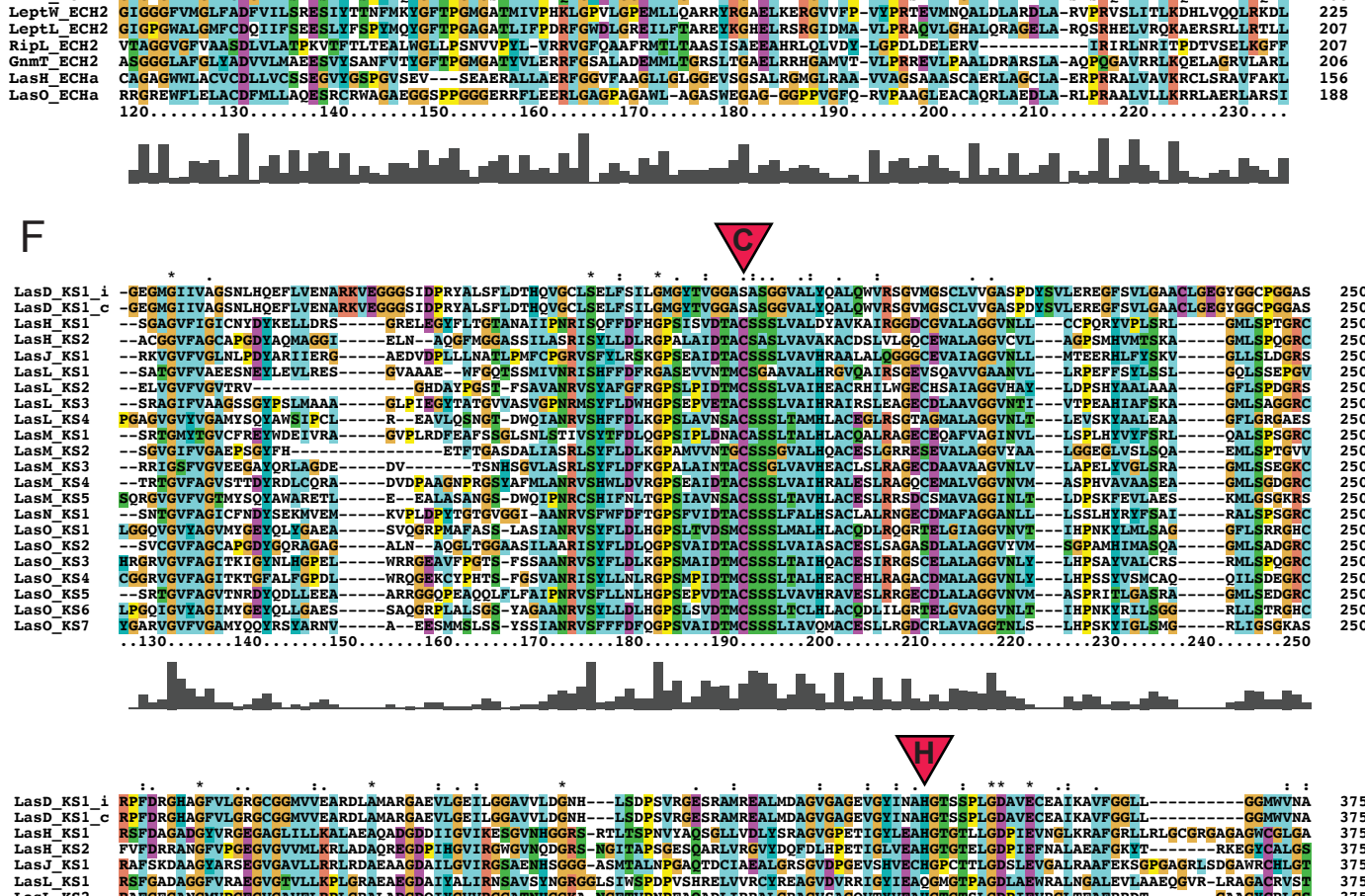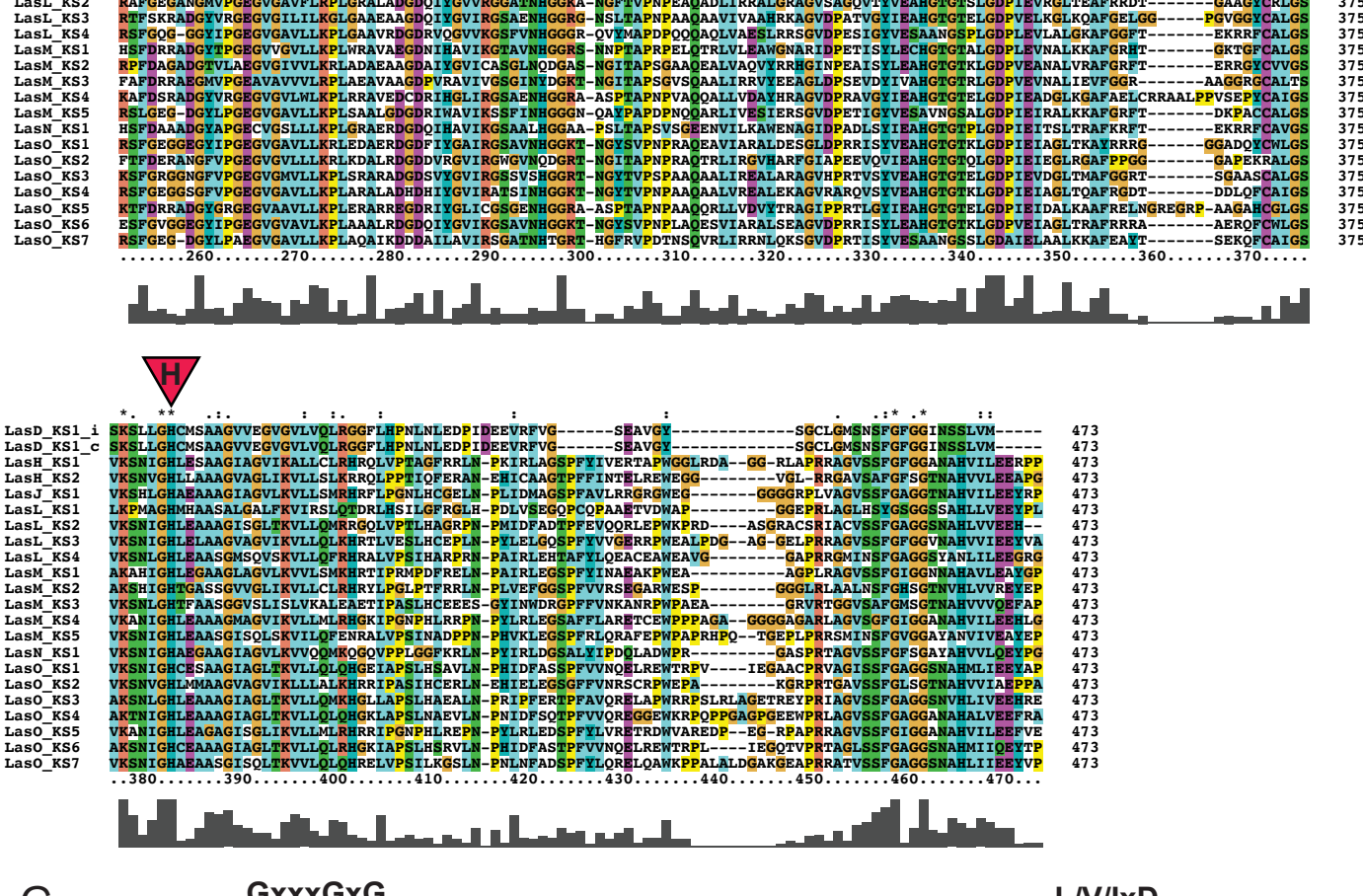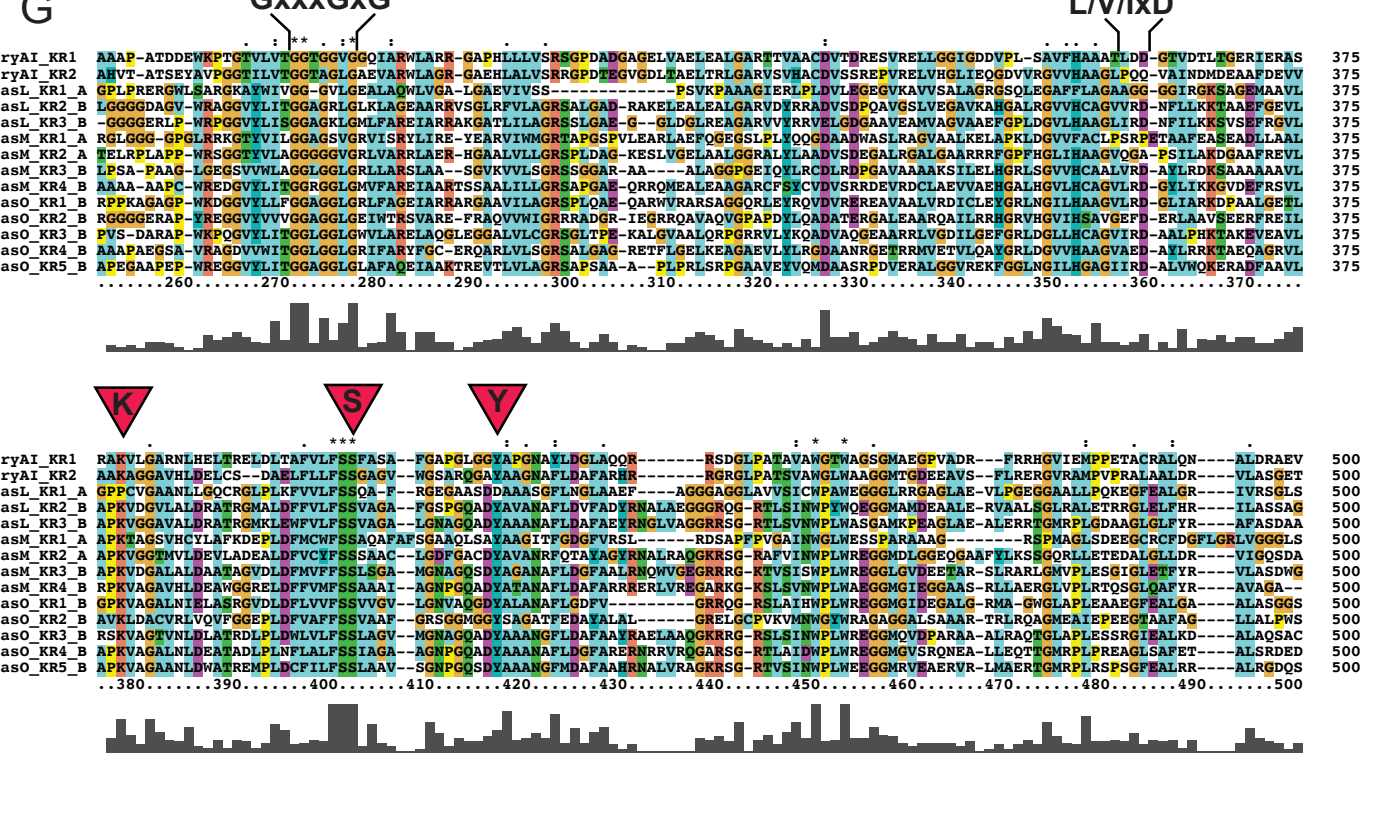

Supplement: DATA SET S2 [file mbio.01524-22-s0006.pdf]

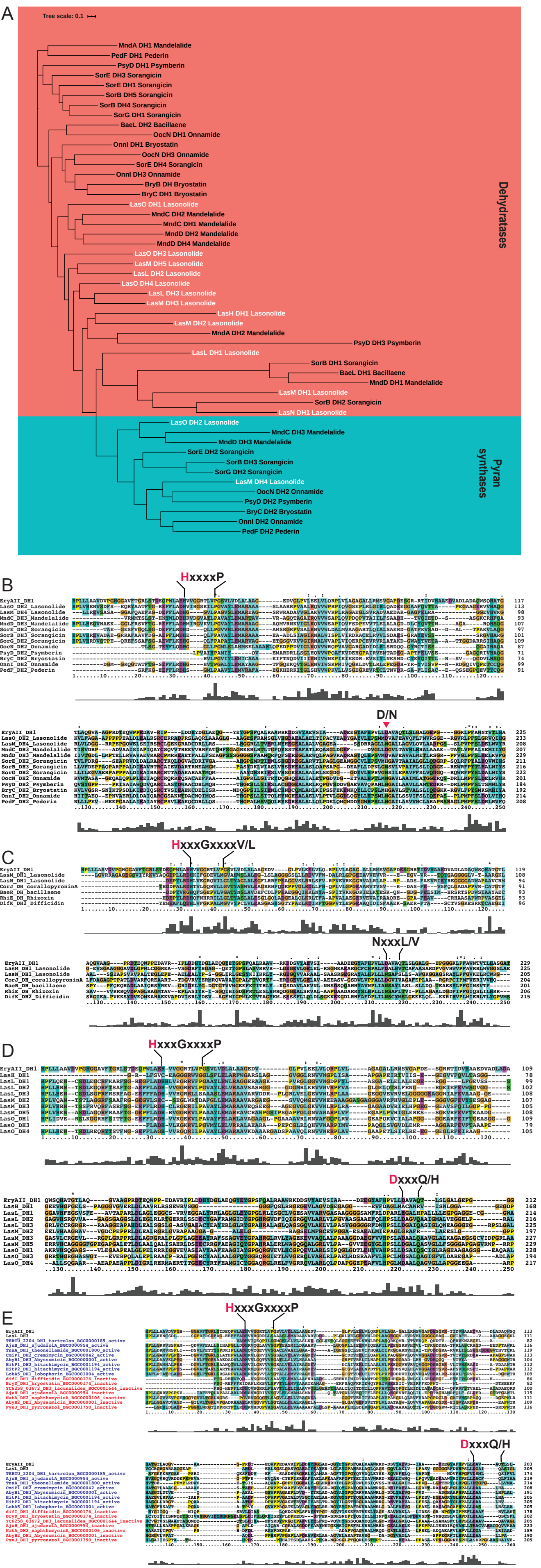

Supplement: DATA SET S3 [file mbio.01524-22-s0007.pdf]
