# Supplementary material for: Uncovering Lasonolide A Biosynthesis Using Genome-Resolved Metagenomics
Source: mBio. 2022 Sep 20;13(5):e01524-22. doi: 10.1128/mbio.01524-22 (PMC9600693; doi:10.1128/mbio.01524-22)
Supplement: FIG S2 [file mbio.01524-22-s0009.pdf]

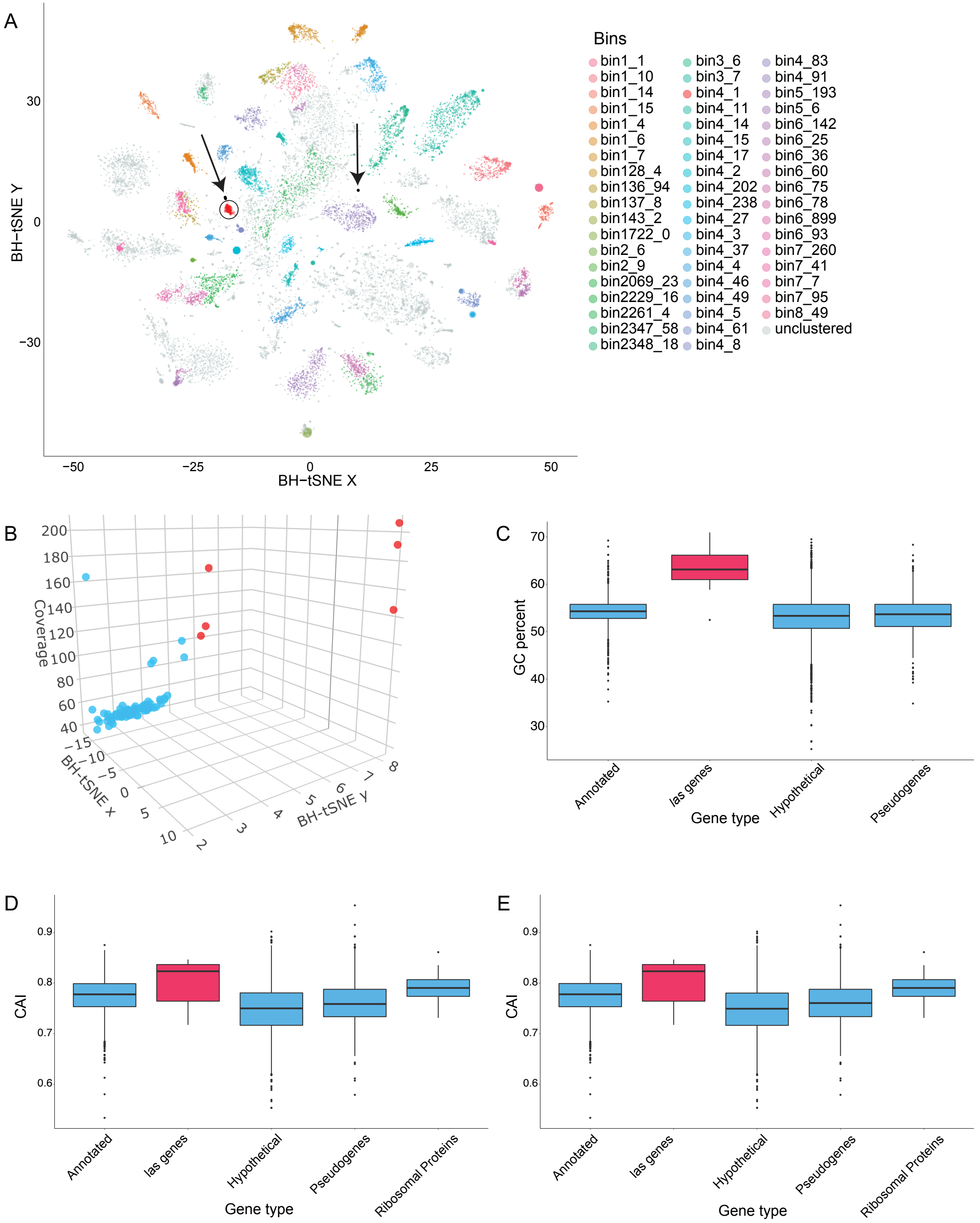


| | Annotated | *las* genes | Hypothetical | Pseudogene | Ribosomal proteins |
|---|---|---|---|---|---|
| Annotated | | 0.0185270 | 0.0000000 | 0.0000000 | 0.1194847 |
| *las* genes | 0.0185270 | | 0.0000001 | 0.0000874 | 0.7082710 |
| Hypothetical | 0.0000000 | 0.0000001 | | 0.0000000 | 0.0000000 |
| Pseudogene | 0.0000000 | 0.0000874 | 0.0000000 | | 0.0000760 |
| Ribosomal proteins | 0.1194847 | 0.7082710 | 0.0000000 | 0.0000760 | |

p-values, ANOVA followed by Tukey HSD

| | Annotated | *las* genes | Hypothetical | Pseudogene | Ribosomal proteins |
|---|---|---|---|---|---|
| Annotated | | 0.0176304 | 0.0000000 | 0.0000000 | 0.1181074 |
| *las* genes | 0.0176304 | | 0.0000001 | 0.0000906 | 0.7018043 |
| Hypothetical | 0.0000000 | 0.0000001 | | 0.0000000 | 0.0000000 |
| Pseudogene | 0.0000000 | 0.0000906 | 0.0000000 | | 0.0000867 |
| Ribosomal proteins | 0.1181074 | 0.7018043 | 0.0000000 | 0.0000867 | |

p-values, ANOVA followed by Tukey HSD
